# National and Regional Burden of Hepatitis C in Ethiopia: A Secondary Analysis of the Global Burden of Disease Study 2023

**DOI:** 10.1155/cjgh/2028285

**Published:** 2026-10-21

**Authors:** Alemayehu Abate, Melkamnesh Azage

**Affiliations:** ^1^ Health Research Development Directorate Amhara Public Health Institute P.O. Box 477 Bahir Dar Ethiopia ephi.gov.et; ^2^ Biology Department Bahir Dar University P.O. Box 79 Bahir Dar Ethiopia bdu.edu.et

**Keywords:** DALYs, Ethiopia, global burden of disease, HCV, hepatitis C infection virus, incidence, mortality, prevalence, YLDs, YLLs

## Abstract

**Background:**

Hepatitis C infection virus (HCV) infection is still a major public health concern in Ethiopia, contributing significantly to an increased risk of cirrhosis, hepatocellular carcinoma, and chronic liver disease. This study aimed to assess the national and regional burden of hepatitis C infection in Ethiopia between 2019 and 2023 using findings from the Global Burden of Disease Study 2023.

**Methods:**

A secondary ecological analysis was conducted using population‐level modeled estimates from the Global Burden of Diseases (GBD) 2023 study. The analysis included national and regional estimates from 2019 to 2023. Key metrics assessed were prevalence, incidence, deaths, mortality rates, Years Lived with Disability (YLDs), Years of Life Lost (YLLs), and Disability‐Adjusted Life Years (DALYs). The Cause of Death Ensemble model (CODEm) was used to estimate cause‐specific mortality, and DisMod‐MR 2.1 was used to model nonfatal outcomes. Descriptive statistical analyses were performed, and results were presented using numbers, rates, and percentages. All analyses were conducted using SPSS Version 27.

**Results:**

The prevalence of hepatitis C infection in Ethiopia increased from 1,525,506 (UI): 1,233,554–1,882,997) in 2019 to 1,653,765 (UI: 1,332,884–2,039,588) in 2023, an increase of almost 8.5%. Correspondingly, the age‐standardized prevalence rate (ASPR) increased steadily from 1647 per 100,000 population (UI: 1482–1812) in 2019 to 1750 per 100,000 (UI: 1575–1925) in 2023. The incidence increased from 188,080 (UI: 148,692–240,059) in 2019 to 200,228 (UI: 157,812–258,693) in 2023, increased by 6.5%. The age‐standardized incidence rate (ASIR) followed a consistent upward trajectory, increasing from 250 per 100,000 (UI: 225–275) in 2019 to 264 per 100,000 (UI: 238–290) in 2023. The number of deaths increased from 7019 (UI: 4614–10,075) in 2019 to 7623 (UI: 4879–11,225) in 2023 with an 8.6% increase. The crude mortality rate decreased from 7.26 per 100,000 (UI: 6.39–8.13) in 2019 to 7.13 per 100,000 (UI: 6.27–7.99) in 2023. The age‐standardized mortality rate (ASMR), however, increased from 447 per 100,000 (UI: 393–501) in 2019 to 500 per 100,000 (UI: 440–560) in 2023. DALYs increased from 259,112 (UI: 165,317–381,194) in 2019 to 287,140 (UI: 176,866–437,025) in 2023. The age‐standardized DALY rate inclined from 426 per 100,000 (UI: 383–469) in 2019 to 457 per 100,000 (UI: 411–503) in 2023. The age‐standardized YLL rate increased from 418 per 100,000 (UI: 376–460) in 2019 to 450 per 100,000 (UI: 405–495) in 2023. The age‐standardized YLD rate remained stable, from 7.3 per 100,000 (UI: 6.6–8.0) in 2019 to 7.3 per 100,000 (UI: 6.6–8.0) in 2023. Prevalence and incidence were higher in females than males. The highest prevalence was observed in the 55+ years age group. Incidence was highest among children and adolescents (5–24 years). Regionally, Oromia, Amhara, and Southern Nations, Nationalities, and Peoples’ Region (SNNPs) recorded the highest burden.

**Conclusion:**

The burden of HCV in Ethiopia increased substantially between 2019 and 2023, with premature mortality representing the predominant component of overall health loss. Marked disparities were observed by age, sex, and geographic region. The findings highlight the need to strengthen HCV infection prevention and control.

## 1. Introduction

Hepatitis C infection is a major global health concern and a significant risk factor for cancer and liver cirrhosis [1]. The initial infection stage is typically asymptomatic; the infection progressively decreases and eventually disappears from the bloodstream in 15%–25% of individuals with hepatitis C virus (HCV) infection. 75%–85% of people will usually develop chronic disease [2]. It is estimated that 58–60 million people worldwide have HCV infection, and 1.0–1.3 million new cases are recorded annually [3]. Africa carries a considerable burden of HCV infection, Africa had the highest global regional prevalence, with approximately 5.3% of the population infected [4]. A meta‐analysis of studies from sub‐Saharan Africa estimated an overall HCV infection seroprevalence of 4.17% [5].

Despite the availability of highly effective direct‐acting antiviral (DAA) therapy, HCV infection continues to be a major cause of liver‐related morbidity and mortality, including cirrhosis and hepatocellular carcinoma, accounting for approximately 290,000 deaths yearly [6]. Additionally, the significant burden of chronic hepatitis C infection was emphasized by the fact that it affected almost 3.26 million children [7]. Access to curative treatment for hepatitis C (HCV) with DAAs remains severely constrained in Ethiopia. In most cases, patients must pay for treatment entirely out of pocket. Even when regimens have been made available through targeted access programs, the subsidized cost has historically been approximately $1200 USD for a full 12‐week course.

The prevalence of hepatitis C infection is higher in low‐ and middle‐income countries (LMICs), where healthcare systems often struggle to provide comprehensive screening, timely diagnosis, and access to antiviral therapy [8]. The global distribution of HCV infection is incredibly diverse because of previous healthcare practices, injecting drug use patterns, blood safety measures, and access to preventative and treatment facilities [9].

Sub‐Saharan Africa has a significant but comparatively underreported share of the global HCV infection burden [10]. Hepatitis C infection virus infection is a significant public health concern in Ethiopia with a seroprevalence of 1%–3% [11]. Ethiopia’s HCV infection endemicity is low to moderate, with considerable geographical and demographic differences [12]. The new WHO initiatives aim to eliminate hepatitis C infection by 2030, with an emphasis on reducing new infections and deaths by 90% and 65%, respectively [13]. Ethiopia has begun coordinating its response to hepatitis C infection with the World Health Organization’s Global Health Sector Strategy on hepatitis C infection [14].

Despite numerous studies, significant limitations affect current HCV infection prevalence estimates in Africa. Current estimates are constrained by underdiagnosis, inadequate surveillance, limited use of confirmatory RNA testing, nonrepresentative sampling, and substantial geographical variation. The substantial uncertainty surrounding HCV infection prevalence estimates in Africa may significantly influence government prioritization and investment decisions. Underestimation of the burden may lead to inadequate funding for screening, diagnosis, and treatment services. Furthermore, weak epidemiological data may limit the ability of countries to attract donor support and develop evidence‐based elimination strategies. Strengthening surveillance systems and generating reliable population‐based prevalence estimates are therefore essential for informing policy decisions, optimizing resource allocation, and accelerating progress toward hepatitis C infection elimination.

Despite the high incidence of hepatitis C infection, there is limited comprehensive evidence describing the national and regional burden of the disease in Ethiopia. Therefore, this study aimed to assess the national and regional burden of hepatitis C infection in Ethiopia between 2019 and 2023 using findings from the Global Burden of Disease Study 2023.

## 2. Methodology

### 2.1. Study Area and Design

The study was conducted using the GBD data of Ethiopia. Ethiopia is located in the Eastern part of Africa and is a Federal Democratic Republic country administratively divided into 11 regional states and 2 city administrations. We conducted a systematic investigation of Ethiopia’s Hepatitis C infection–related deaths, mortality rates, Years Lived with Disability (YLDs), Years of Life Lost (YLLs), and Disability‐Adjusted Life Years (DALYs) from the 2023 Global Burden of Diseases, Injuries, and Risk Factors Study (GBD [15]). The Cause of Death Ensemble model (CODEm) was used by GBD to model cause‐specific Metrix [16]. GBD estimate used DisMod‐MR 2.1 to model nonfatal outcomes [17], and the GBD estimate used hierarchical cause list and covariate selection framework to ensure internal consistency across estimates [18].

### 2.2. Inclusion and Exclusion Criteria

Data were included in the analysis if they met the following criteria: data specific to Ethiopia at national and regional estimates covering the period 2019–2023. Estimates prior to 2019 of GBD 2023 outputs were excluded from the study.

### 2.3. Operational Definitions

HCV infection: a GBD‐defined health condition representing the population‐level burden attributable to HCV infection, as estimated by the Global Burden of Disease Study 2023. In this study, the term refers specifically to the HCV infection outcome defined within the GBD framework and is not used interchangeably with anti‐HCV infection antibody positivity or laboratory‐confirmed active/viremic infection.

HCV infection prevalence: The estimated proportion of the population living with the GBD‐defined HCV infection outcome at a specified time. Prevalence estimates are reported as the number of prevalent cases and as a rate per 100,000 population. The GBD‐modeled prevalence should not be interpreted as directly equivalent to anti‐HCV infection seroprevalence from population‐based serological surveys.

HCV infection incidence: The estimated number of new HCV infection cases occurring in the population during a specified period, according to the GBD 2023 modeling framework. Incidence is reported as the number of incident cases and as a rate per 100,000 population and should not be interpreted as equivalent to newly detected anti‐HCV infection antibody positivity.

HCV infection–related mortality: The number of deaths attributable to HCV infection according to the GBD cause‐of‐death framework. Mortality is reported as the number of deaths and as a rate per 100,000 population. It represents HCV infection–attributable mortality within the GBD framework rather than deaths restricted to acute hepatitis C infection.

Disability‐adjusted life years (DALYs): A summary measure of overall health loss attributable to HCV infection, calculated as the sum of years of life lost due to premature mortality (YLLs) and years lived with disability (YLDs): DALY = YLL + YLD.

Years of life lost (YLLs): YLLs because of premature death attributable to HCV infection, estimated according to the GBD standard life expectancy.

Years lived with disability (YLDs): Years lived in less than full health because of HCV infection–related morbidity, weighted according to the severity of the associated health states in the GBD framework.

Crude rate: The number of HCV infection cases, incident cases, deaths, or DALYs divided by the corresponding population and multiplied by 100,000. It reflects the actual population‐level burden without adjustment for differences in population age structure.

Age‐standardized rate (ASR): A summary rate adjusted for differences in population age structure using the selected standard population. It was calculated by direct standardization as the weighted sum of age‐specific rates: ASR = Σ (*r*_*i*_ × *w*_*i*_), where *r*_*i*_ is the age‐specific rate for age group *i* and *w*_*i*_ is the corresponding proportion of the standard population.

Sex‐specific rate: The HCV infection prevalence, incidence, mortality, or DALY rate calculated separately for males and females, expressed per 100,000 population.

Absolute burden: The total number of prevalent cases, incident cases, deaths, or DALYs attributed to HCV infection. Absolute numbers were interpreted as indicators of the magnitude of healthcare and resource requirements rather than direct measures of population‐level risk.

Rate ratio (RR): The ratio of the HCV infection rate in one population group to that of a reference group. For example, the sex‐specific rate ratio was calculated as the rate among males divided by the rate among females, or vice versa, depending on the specified reference group.

Uncertainty interval (UI): The 95% UI represents the range containing the uncertainty around the GBD point estimate based on the GBD modeling framework and its underlying posterior distributions/draws. It reflects uncertainty arising from the available data and modeling processes and is not equivalent to a conventional 95% confidence interval.

Harm reduction policies and practices: In this study, harm reduction refers to public health policies and targeted interventions intended to reduce HCV transmission through exposure to infected blood, including prevention of unsafe injections, provision of safe injection practices and equipment, blood‐safety measures, infection‐prevention and control practices, and other interventions targeting populations with increased exposure risk. In the Ethiopian context, these interventions are considered within the existing hepatitis response and phased public health microelimination approach.

### 2.4. Geographical and Temporal Scope

National Level: The analysis provides estimates for the Federal Democratic Republic of Ethiopia as a whole. Regional Level: Estimates were generated for the regional states of Federal Democratic Republic of Ethiopia estimates corresponded to the GBD‐defined regional states and city administrations available in the GBD Results Tool. Temporal Frame: The primary period of analysis for this paper is 2019–2023. The models, however, leverage data from 1990 onwards to ensure robust trend estimation and to enhance the predictive validity of the 2019–2023 estimates.

### 2.5. Data Sources and Identification

Data on hepatitis C infection virus in Ethiopia was extracted from GBD 2023. The study’s data can be accessed from the Global Health Data Exchange GBD Results Tool (https://ghdx.healthdata.org/gbd-results-tool). Data were extracted based on the study’s requirements by age, sex, and location. The GBD 2023 population database, which compiles information from censuses, demographic surveillance sites, and the World Population Prospects, provided the population estimates for Ethiopia and its subnational regions.

### 2.6. GBD Data Extraction and Definitions

Data for this study were obtained from the Global Burden of Disease Study 2023 (GBD 2023) Results Tool to estimate the burden of hepatitis C infection in Ethiopia and its subnational locations. The extraction was conducted using the GBD‐defined cause “Total burden related to hepatitis C infection” (cause ID 1027). The analysis included four principal measures: deaths (measure ID 1), DALYs (measure ID 2), prevalence (measure ID 5), and incidence (measure ID 6). Additional GBD components of DALYs, including YLDs (measure ID 3) and YLLs (measure ID 4), were also considered where applicable.

The GBD data were extracted using three metrics: number (metric ID 1), percent (metric ID 2), and rate (metric ID 3). Number estimates were used to quantify the absolute magnitude of HCV infection burden, while rate estimates were used for comparisons between populations with different population sizes and demographic structures. Percentage estimates were used where appropriate to describe the proportional contribution of HCV infection–related outcomes.

The analysis covered Ethiopia and its GBD‐defined subnational locations, including Tigray (location ID 44852), Afar (44853), Amhara (44854), Oromia (44855), Somali (44856), Benishangul‐Gumuz (44857), Harari (44859), Gambella (44860), Addis Ababa (44861), Dire Dawa (44862), Southern Nations, Nationalities, and Peoples (SNNPs; 95069), Sidama (60908), and South West (94364). The GBD geographical hierarchy was used without independently modifying administrative boundaries. Thus, estimates for locations affected by administrative reorganization were interpreted according to the geographical definitions used in GBD 2023.

Estimates were extracted by sex, including male (sex ID 1), female (sex ID 2), and both sexes where applicable. Age‐specific estimates included < 5 years (age ID 1), 15–19 years (age ID 8), 20–24 years (age ID 9), 25–29 years (age ID 10), 30–34 years (age ID 11), 35–39 years (age ID 12), 40–44 years (age ID 13), 45–49 years (age ID 14), 50–54 years (age ID 15), 5–14 years (age ID 23), and ≥ 55 years (age ID 228). These age categories were retained as defined by the GBD Results Tool to ensure consistency and reproducibility.

### 2.7. Population and Demographic Data

The GBD 2023 population database, which compiles information from censuses, demographic surveillance sites, and the UN World Population Prospects, provided the population estimates for Ethiopia and its regions, including age and sex distributions. The Cause of Death Ensemble model (CODEm), an efficient algorithmic tool that evaluates a large number of credible statistical models and chooses the top‐performing combination based on out‐of‐sample predictive validity, was used to predict hepatitis C infection mortality.

### 2.8. Statistical Analysis

The extracted dataset was reviewed to ensure that each predefined combination of location, year, age group, sex, measure, and metric was uniquely represented. No observations were duplicated in the final analytic dataset. Where applicable, location, age, sex, measure, and metric labels were standardized according to the GBD location and variable definitions rather than manually redefining the underlying estimates. No estimates were imputed or statistically replaced by the authors.

A comprehensive analysis was conducted into the prevalence, incidence, deaths, mortality rate, adjusted life years, YLLs, and YLDs of HCV infection. Data of HCV infection were assessed by age, sex, and location from the GBD study. For each study year, age group, sex, and region, the number of cases and corresponding rates were summarized. Where applicable, rates were calculated as: Rate = (Number of events/Population) × 100,000. The percentage change between 2019 and 2023 was calculated as Percentage change = [(Estimate in 2023 − Estimate in 2019)/Estimate in 2019] × 100. ASRs were calculated using the direct standardization method by applying age‐specific rates to the corresponding weights of the standard population: ASR = *Σ*(*r*_*i*_ × *w*_*i*_)/*Σ* *w*_*i*_, where *r*_*i*_ represents the age‐specific rate for age group *i*, and *w*_*i*_ represents the corresponding standard population weight.

For GBD estimates, uncertainty was expressed using 95% UIs, defined by the 2.5th and 97.5th percentiles of the GBD posterior distribution. Point estimates and corresponding 95% UIs were reported throughout the main tables and Results section.

Temporal changes were assessed descriptively by calculating the percentage change between the first and last years of the study period. Because a formal log‐linear regression model was not applied, these estimates were interpreted as endpoint changes rather than formal estimates of annual temporal trends. Regional, age‐, and sex‐specific estimates were compared descriptively using point estimates and their corresponding 95% UIs. The study followed the recommendations of the STROBE guideline [19] and GATHER guideline [20].

### 2.9. Ethical Considerations

The study was exempt from the institutional review board approval since it used aggregated, anonymized data from databases and publicly accessible sources. Every original source of data used in the GBD study complies with regional ethical standards.

## 3. Results

### 3.1. Prevalence and Incidence of Hepatitis C Infection in Ethiopia (2019–2023)

The prevalence of hepatitis C infection in Ethiopia increased from 1,525,506 (95% UI: 1,233,554–1,882,997) in 2019 to 1,653,765 (UI: 1,332,884–2,039,588) in 2023. Correspondingly, the age‐standardized prevalence rate (ASPR) increased steadily from 1647 per 100,000 population (UI: 1482–1812) in 2019 to 1750 per 100,000 (UI: 1575–1925) in 2023.

The incidence increased from 188,080 (UI: 148,692–240,059) in 2019 to 200,228 (UI: 157,812–258,693) in 2023. The age‐standardized incidence rate (ASIR) followed a consistent upward trajectory, increasing from 250 per 100,000 (UI: 225–275) in 2019 to 264 per 100,000 (UI: 238–290) in 2023.

### 3.2. Deaths and Mortality of Hepatitis C Infection in Ethiopia (2019–2023)

The number of deaths increased from 7019 (UI: 4614–10,075) in 2019 to 7623 (UI: 4879–11,225) in 2023. with 8.6% increase. The crude mortality rate decreased from 7.26 per 100,000 (UI: 6.39–8.13) in 2019 to 7.13 per 100,000 (UI: 6.27–7.99) in 2023. The age‐standardized mortality rate (ASMR), however, increased from 447 per 100,000 (UI: 393–501) in 2019 to 500 per 100,000 (UI: 440–560) in 2023.

### 3.3. DALYs, YLLs, and YLDs of Hepatitis C Infection in Ethiopia (2019–2023)

DALYs increased from 259,112 (UI: 165,317–381,194) in 2019 to 287,140 (UI: 176,866–437,025) in 2023. The age‐standardized DALY rate inclined from 426 per 100,000 (UI: 383–469) in 2019 to 457 per 100,000 (UI: 411–503) in 2023. YLLs increased from 254,596 (UI: 161,246–375,627) in 2019 to 282,415 (UI: 172,238–432,101) in 2023. The age‐standardized YLL rate increased from 418 per 100,000 (UI: 376–460) in 2019 to 450 per 100,000 (UI: 405–495) in 2023. In contrast, YLDs remained relatively stable over the study period, increasing slightly from 4516 (UI: 2681–7170) in 2019 to 4725 (UI: 2763–7530) in 2023. The age‐standardized YLD rate remained stable, from 7.3 per 100,000 (UI: 6.6–8.0) in 2019 to 7.3 per 100,000 (UI: 6.6–8.0) in 2023, with a small decline to 7.1 per 100,000 (UI: 6.4–7.8) in 2022 (Table 1 and Annex 1).

**TABLE 1:** Hepatitis C infection burden and temporal trends in Ethiopia, 2019–2023.

Measure	2019	2020	2021	2022	2023
Prevalence	1,525,506	1,553,900	1,584,972	1,621,349	1,653,765
Prevalence‐age‐standardized rate	1647	1671	1697	1724	1750
Incidence	188,080	190,612	193,744	197,423	200,228
Incidence‐age‐standardized rate	250	253	257	261	264
Total deaths	7019	6842	6675	7168	7623
Mortality rate	7.26	6.93	6.58	6.97	7.13
Mortality rate‐age‐standardized rate	447	428	438	466	500
DALYs	259,112	253,836	247,590	268,648	287,140
DALYs‐age‐standardized rate	426	419	408	434	457
YLLs	254,596	249,338	243,081	264,086	282,415
YLLs‐age‐standardized rate	418	412	401	427	450
YLDs	4516	4498	4508	4562	4725
YLDs‐age‐standardized rate	7.3	7.2	7.2	7.1	7.3

### 3.4. Age and Sex Specific in Incidence and Prevalence of Hepatitis C Infection in Ethiopia, 2019–2023

The prevalence and incidence of hepatitis C infection was higher in females than males. The prevalence of hepatitis C infection increased among females from 779,145 (UI: 634,869–951,012) in 2019 to 857,216 (UI: 700,453–1,046,893) in 2023. Among males, prevalence increased from 746,361 (UI: 598,685–931,985) in 2019 to 796,549 (UI: 632,431–992,695) in 2023, The prevalence rate among females increased from 1438 (UI: 1435–1441) to 1446 (UI: 1443–1449) per 100,000, whereas the male prevalence rate declined from 1387 (UI: 1384–1390) per 100,000 in 2019 to 1355 (UI: 1352–1358) per 100,000 in 2023. Consequently, the male‐to‐female prevalence rate ratio decreased from 0.965 (UI: 0.963–0.967) in 2019 to 0.937 (UI: 0.935–0.939) in 2023.

A similar female predominance was observed for incidence. Incident cases increased from 95,145 (UI: 76,309–119,983) to 102,489 (UI: 81,956–131,675) among females and from 92,935 (UI: 72,383–120,076) to 97,739 (UI: 75,856–127,018) among males between 2019 and 2023. The incidence rates decreased among females, from 176 (UI: 175–177) in 2019 to173 (UI: 172–174) in 2023. The incident rate among males also declined from 173 (UI: 172–174) in 2019 to 166 (UI: 165–167) in 2023. The male‐to‐female incidence rate ratio remained below 1 throughout the study period, ranging from 0.959 to 0.984.

The prevalence increased with increasing age, The highest prevalence was recorded in the 55+ years age group, increasing from 387,512 (UI: 314,694–475,845) in 2019 to 418,649 (UI: 342,034–511,194) in 2023. This was followed by individuals aged 35–44 years and 25–34 years, The prevalence in the 35–44 years age group increased from 268,545 (UI: 215,942–340,072) in 2019 to 299,162 (UI: 239,586–377,267) in 2023. The prevalence in the 25–34 years age group increased from 263994 (UI: 214514–322555) in 2019–288171 (UI: 230902–355194) in 2023, However, in prevalence rate, the 45–54‐year group recorded the highest prevalence rate of any stratum, rising from 6682 (UI: 6655–6709) to 7515 (UI: 7486–7544) per 100,000 an increase of 12.5% over the observation period.

The incidence of hepatitis C infection was high among children aged 5–14 years which from 35,821 (UI: 30,131–42,551) in 2019 to 36,278 (UI: 30,285–43,643) in 2023. The incidence of hepatitis C infection among children aged < 5 years increased from 16,012 (UI: 13,049–19,179) in 2019 to 16,428 (UI: 13,205–19,555) in 2023.

Age‐stratified incidence rate trends by age group were consistent with the prevalence patterns observed above, with the steepest increases concentrated in working‐age adults. The 45–54‐year group recorded the highest incidence rate of any age stratum across all years, rising from 660 (UI: 654–666) in 2019 to 735 (UI: 729–741) per 100,000 in 2023 (Table 2 and Annex 2).

**TABLE 2:** Age and sex specific incidence and prevalence of hepatitis C infection in Ethiopia, 2019–2023.

Measure	Category	2019	2020	2021	2022	2023
Prevalence (cases)	Female	779,145	796,193	817,311	840,858	857,216
	Male	746,361	757,707	767,661	780,491	796,549

Prevalence rate	Female	1438	1437	1441	1450	1446
	Male	1387	1378	1366	1357	1355
	Male‐to‐female ratio	0.965	0.959	0.948	0.936	0.937

Incidence (cases)	Female	95,145	96,645	98,656	100,938	102,489
	Male	92,935	93,967	95,089	96,485	97,739

Incidence rate	Female	176	174	174	174	173
	Male	173	171	169	168	166
	Male‐to‐female ratio	0.984	0.959	0.972	0.964	0.962

Prevalence (cases)	< 5 years	16,125	16,191	16,279	16,400	16,493
	5–14 years	127,034	127,081	127,382	127,961	128,272
	15–24 years	226,373	228,959	231,757	234,907	237,632
	25–34 years	263,994	269,399	275,699	282,656	288,171
	35–44 years	268,545	275,458	283,175	291,572	299,162
	45–54 years	235,923	242,162	249,632	258,080	265,386
	55+ years	387,512	394,650	401,049	409,774	418,649

Prevalence rate	< 5 years	215.00	215.88	217.05	218.67	219.91
	5–14 years	705.74	706.01	707.68	710.89	712.62
	15–24 years	2791	2822	2856	2895	2930
	25–34 years	3867	3945	4038	4140	4224
	35–44 years	5048	5178	5323	5481	5628
	45–54 years	6682	6859	7070	7309	7515
	55+ years	4079.00	4154.00	4221.00	4313.00	4406.83

Incidence (cases)	< 5 years	16,012	16,063	16,131	16,272	16,428
	5–14 years	35,821	35,832	35,939	36,138	36,278
	15–24 years	34,920	35,269	35,673	36,114	36,451
	25–34 years	30,305	30,835	31,511	32,263	32,842
	35–44 years	28,561	29,165	29,891	30,695	31,440
	45–54 years	23,484	24,078	24,844	25,699	26,181
	55+ years	18,977	19,369	19,755	20,242	20,608

Incidence rate	< 5 years	213	214	215	217	219
	5–14 years	413	413	415	418	421
	15–24 years	429	433	438	443	447
	25–34 years	443	451	461	472	480
	35–44 years	537	548	562	577	592
	45–54 years	660	676	698	722	735
	55+ years	200	204	208	213	217

### 3.5. Regional Trend Analysis of Prevalence and Incidence of Hepatitis C Infection (2019–2023)

In Amhara, the prevalence of hepatitis C infection increased from 360060 (UI: 287012–447614) in 2019–384282 (UI: 304106–480606) in 2023. In Oromia, the prevalence of hepatitis C infection increased from 499373 (UI: 387545–630079) in 2019–551992 (UI: 424697–706695) in 2023. In SNNPs, the prevalence of hepatitis C infection increased from 242477 (UI: 197577–297173) in 2019–267385 (UI: 215016–328882) in 2023.

In Amhara, incidence increased from 41,130 (UI: 31,659–53,346) in 2019 to 42,928 (UI: 32,401–56,532) in 2023. In Oromia, the incidence increased from 65,410 (UI: 48,855–86,341) in 2019 to 71,266 (UI: 53,048–96,177) in 2023. In SNNPs, the incidence increased from 28,924 (UI: 22,706–37,035) in 2019 to 31,168 (UI: 23,979–40,321) in 2023.

### 3.6. Regional Trend Analysis of Deaths and Mortality of Hepatitis C Infection (2019–2023)

Oromia and Amhara had the largest absolute number of deaths among larger, more populous regions. The number of deaths in Oromia increased from 2444 (UI: 1494–3664) in 2019 to 2716 (UI: 1605–4302) in 2023. The number of deaths in Amhara increased from 1518 (UI: 968–2252) in 2019 to 1631 (UI: 1003–2451) in 2023. The ASPR was consistently highest in Oromia, increasing from 42,981.6 (UI: 39,441.9–46,769.5) per 100,000 in 2019 to 46,693.0 (UI: 42,730.2–50,975.2) in 2023. Amhara had the second‐highest ASPR, increasing from 28,523.1 (UI: 26,238.2–30,948.1) to 29,668.7 (UI: 27,238.2–32,272.5) during the same period.

The ASIR showed a similar marked regional gradient. Oromia had the highest ASIR throughout the study period, increasing from 6590.2 (UI: 6115.7–7098.6) per 100,000 in 2019 to 7069.5 (UI: 6544.3–7640.7) in 2023. The rate in Amhara increased from 3904.6 (UI: 3619.8–4198.2) to 4015.8 (UI: 3713.9–4337.6), while SNNPs increased from 3125.9 (UI: 2901.5–3363.7) to 3331.5 (UI: 3086.2–3597.5).

Regional mortality rates displayed a different pattern from prevalence and incidence. SNNPs recorded the highest ASMR throughout the study period, declining initially from 12.35 (UI: 11.17–13.53) per 100,000 in 2019 to 11.70 (UI: 10.58–12.82) in 2021, before increasing substantially to 13.87 (UI: 12.55–15.19) in 2023 (Table 3 and Annex 3).

**TABLE 3:** Regional prevalence, incidence, deaths, and mortality rate trends of hepatitis C infection in Ethiopia, 2019–2023.

Measure	Region	2019	2020	2021	2022	2023
Prevalence	Addis Ababa	60,158	60,839	61,235	61,968	62,696
	Afar	28,236	28,451	28,492	28,683	28,979
	Amhara	360,060	364,698	371,012	378,512	384,282
	Benishangul‐Gumuz	20,841	21,477	22,228	23,060	23,838
	Dire Dawa	7163	7256	7333	7442	7532
	Gambella	7010	7162	7281	7441	7600
	Harari	5014	5088	5091	5123	5199
	Oromia	499,373	510,220	522,221	536,293	551,992
	Sidama	66,650	68,047	69,575	71,230	72,126
	Somali	96,221	97,855	99,188	101,059	103,545
	South West	39,795	40,133	40,414	40,828	41,344
	SNNPs	242,477	248,944	255,902	263,225	267,385
	Tigray	92,509	93,731	95,001	96,487	97,247

Incidence	Addis Ababa	6655	6683	6689	6714	6719
	Afar	3860	3873	3873	3888	3904
	Amhara	41,130	41,398	41,903	42,534	42,928
	Benishangul‐Gumuz	2798	2868	2951	3044	3128
	Dire Dawa	844	849	854	862	866
	Gambella	895	907	918	933	942
	Harari	625	631	632	634	637
	Oromia	65,410	66,566	67,998	69,661	71,266
	Sidama	8551	8679	8831	8997	9063
	Somali	12,587	12,747	12,901	13,106	13,344
	South West	5128	5143	5163	5196	5217
	SNNPs	28,924	29,503	30,161	30,856	31,168
	Tigray	10,670	10,764	10,869	10,997	11,047

Deaths (total number)	Addis Ababa	393	384	387	403	425
	Afar	118	118	116	118	126
	Amhara	1518	1482	1443	1539	1631
	Benishangul‐Gumuz	89	88	88	90	95
	Dire Dawa	35	35	34	35	36
	Gambella	30	30	30	31	34
	Harari	19	18	18	19	20
	Oromia	2444	2378	2316	2550	2716
	Sidama	323	311	297	320	338
	Somali	414	409	403	419	446
	South West	235	227	214	220	230
	SNNPs	994	962	942	1028	1111
	Tigray	407	401	388	395	413

Mortality Rate	Addis Ababa	306	291	292	298	306
	Afar	312	303	296	300	312
	Amhara	209	198	187	196	202
	Benishangul‐Gumuz	286	269	255	249	251
	Dire Dawa	238	227	215	216	219
	Gambella	250	242	234	238	251
	Harari	247	229	224	235	240
	Oromia	265	249	236	255	262
	Sidama	326	302	280	295	300
	Somali	265	252	240	247	253
	South West	385	363	333	339	344
	SNNPs	247	229	216	229	238
	Tigray	209	200	188	187	189

ASR—prevalence	Addis Ababa	4958.00	4986.30	5026.30	5080.20	5106.40
	Afar	2471.30	2496.50	2510.40	2531.80	2549.10
	Amhara	28,523.10	28,729.60	29,032.50	29,390.40	29,668.70
	Benishangul‐Gumuz	1900.30	1946.50	1999.50	2055.00	2104.90
	Dire Dawa	523.6	526.1	528.9	532	531.2
	Gambella	641.9	648.1	653.9	658.6	662.1
	Harari	404.3	407.5	408.1	409.6	412.1
	Oromia	42,981.60	43,812.20	44,708.10	45,685.50	46,693.00
	Sidama	6813.70	6902.90	7005.40	7113.00	7159.00
	Somali	8942.40	9092.60	9228.50	9395.80	9587.10
	South West	3872.20	3898.20	3919.70	3945.80	3972.10
	SNNPs	21,820.60	22,238.60	22,694.30	23,159.50	23,349.70
	Tigray	7981.80	8047.80	8109.70	8187.40	8225.90

ASR—Incidence	Addis Ababa	558.1	557.6	555	554.6	553.9
	Afar	378.6	380.5	381.3	383	384.4
	Amhara	3904.60	3919.60	3948.90	3992.10	4015.80
	Benishangul‐Gumuz	294.5	299.7	306.1	312.8	319
	Dire Dawa	82.5	82.4	82.4	82.6	82.5
	Gambella	99.1	99.6	100.2	100.7	100.8
	Harari	60.2	60.6	60.5	60.6	60.9
	Oromia	6590.20	6674.70	6785.80	6917.40	7069.50
	Sidama	968.5	979.4	993.2	1008.20	1012.50
	Somali	1411.00	1425.10	1440.80	1460.70	1482.50
	South West	517.1	519	521.3	524.4	526.1
	SNNPs	3125.90	3181.10	3242.80	3304.60	3331.50
	Tigray	1165.60	1171.00	1176.50	1185.80	1189.10

ASR—mortality rate	Addis Ababa	11.52	11.24	11.34	11.8	12.45
	Afar	8.35	8.35	8.21	8.35	8.92
	Amhara	10.14	9.9	9.64	10.27	10.87
	Benishangul‐Gumuz	10.53	10.41	10.41	10.65	11.23
	Dire Dawa	9.55	9.55	9.27	9.55	9.87
	Gambella	8.93	8.93	8.93	9.23	10.12
	Harari	10.02	9.5	9.5	10.02	10.56
	Oromia	8.97	8.72	8.49	9.35	9.94
	Sidama	9.12	8.8	8.4	9.06	9.56
	Somali	8.17	8.06	7.96	8.26	8.78
	South West	8.62	8.32	7.85	8.07	8.45
	SNNPs	12.35	11.95	11.7	12.83	13.87
	Tigray	10.59	10.44	10.1	10.27	11.45

### 3.7. Regional DALYs, YLLs, and YLDs of Hepatitis C Infection 2019–2023

The DALYs in Oromia increased from 89,919 (UI: 52,103–139,297) in 2019 to 103,076 (UI: 56,804–168,771) in 2023. The DALYs in Amhara increased from 50,825 (UI: 31,916–77,274) in 2019 to 55,470 (UI: 32,697–87,198) in 2023. The DALYs in SNNPs increased from 38,176 (UI: 23,065–58,496) in 2019 to 43,050 (UI: 24,642–69,936) in 2023. In Oromia, the YLLs increased from 88,471 in 2019 to 101,502 in 2023. In Amhara, the YLLs increased from 49,886 in 2019 to 54,495 in 2023.

SNNPs had the highest ASR‐DALY in 2019 (1542; 95% UI: 1527–1557), followed by Amhara (1523; 95% UI: 1510–1536), while South West had the lowest (908; 95% UI: 889–927). By 2023, SNNPs remained the highest at 1739 (95% UI: 1722–1756), followed by Amhara at 1662 (95% UI: 1649–1675).

The age‐standardized YLL rates followed a similar temporal pattern to ASR‐DALYs. SNNPs had the highest rate in 2019 (1511; 95% UI: 1496–1526) and 2023 (1707; 95% UI: 1690–1724. Age‐standardized YLD rates were also relatively stable compared with YLL rates. The highest ASR‐YLD was consistently observed in Harari, ranging from 37.4 (95% UI: 22.8–52.0) in 2019 to 37.6 (95% UI: 22.9–52.3) in 2023 (Table 4 and Annex 4).

**TABLE 4:** Regional YLLs, YLDs, and DALYs of hepatitis C infection in Ethiopia, 2019–2023.

Region	Measure	2019	2020	2021	2022	2023
Addis Ababa	DALYs (Crude)	15,845	15,548	15,679	16,528	17,388
	ASR_DALYs	1248	1225	1235	1302	1370
	YLLs (Crude)	15,625	15,331	15,468	16,324	17,176
	ASR_YLLs	1231	1208	1218	1286	1353
	YLDs (Crude)	221	218	211	204	212
	ASR_YLDs	17.4	17.2	16.6	16.1	16.7

Afar	DALYs (Crude)	4811	4808	4801	4932	5288
	ASR_DALYs	958	958	957	983	1054
	YLLs (Crude)	4721	4719	4712	4843	5196
	ASR_YLLs	941	940	939	965	1035
	YLDs (Crude)	90	89	88	89	92
	ASR_YLDs	17.9	17.8	17.6	17.7	18.3

Amhara	DALYs (Crude)	50,825	49,748	48,315	52,056	55,470
	ASR_DALYs	1523	1490	1447	1559	1662
	YLLs (Crude)	49,886	48,823	47,376	51,104	54,495
	ASR_YLLs	1495	1463	1419	1531	1633
	YLDs (Crude)	939	925	939	952	975
	ASR_YLDs	28.1	27.7	28	28.4	29.1

Benishangul‐Gumuz	DALYs (Crude)	3660	3621	3581	3702	3928
	ASR_DALYs	1314	1300	1285	1329	1410
	YLLs (Crude)	3593	3553	3511	3632	3855
	ASR_YLLs	1290	1276	1261	1304	1384
	YLDs (Crude)	68	69	69	70	74
	ASR_YLDs	24.4	24.7	24.7	25.1	26.3

Dire Dawa	DALYs (Crude)	1334	1326	1275	1324	1388
	ASR_DALYs	1045	1039	999	1037	1088
	YLLs (Crude)	1313	1306	1255	1304	1368
	ASR_YLLs	1029	1023	983	1021	1072
	YLDs (Crude)	20	20	20	20	20
	ASR_YLDs	15.7	15.8	15.5	15.8	15.7

Gambella	DALYs (Crude)	1235	1263	1251	1310	1444
	ASR_DALYs	1098	1123	1112	1164	1283
	YLLs (Crude)	1214	1241	1230	1289	1422
	ASR_YLLs	1079	1104	1094	1146	1264
	YLDs (Crude)	21	21	21	21	22
	ASR_YLDs	18.7	19	18.5	18.6	19.2

Harari	DALYs (Crude)	728	704	704	752	792
	ASR_DALYs	1182	1143	1143	1220	1286
	YLLs (Crude)	705	681	682	730	769
	ASR_YLLs	1144	1105	1108	1185	1248
	YLDs (Crude)	23	23	22	22	23
	ASR_YLDs	37.4	37.7	36.1	36.2	37.6

Oromia	DALYs (Crude)	89,919	88,075	86,032	96,002	######
	ASR_DALYs	1096	1073	1048	1170	1256
	YLLs (Crude)	88,471	86,627	84,571	94,502	######
	ASR_YLLs	1079	1056	1031	1152	1237
	YLDs (Crude)	1448	1449	1461	1500	1574
	ASR_YLDs	17.7	17.7	17.6	17.8	18.9

Sidama	DALYs (Crude)	12,615	12,237	11,686	12,718	13,495
	ASR_DALYs	1043	1012	966	1052	1116
	YLLs (Crude)	12,350	11,970	11,421	12,455	13,231
	ASR_YLLs	1021	990	944	1030	1094
	YLDs (Crude)	266	267	264	262	264
	ASR_YLDs	22	22.2	22	21.8	21.8

Somali	DALYs (Crude)	16,433	16,268	15,991	16,869	17,980
	ASR_DALYs	1162	1150	1131	1193	1272
	YLLs (Crude)	16,157	15,992	15,716	16,592	17,689
	ASR_YLLs	1143	1131	1111	1173	1251
	YLDs (Crude)	275	276	275	277	290
	ASR_YLDs	19.5	19.5	19.7	19.5	20.5

South West	DALYs (Crude)	9486	9259	8688	8993	9405
	ASR_DALYs	908	886	832	861	900
	YLLs (Crude)	9352	9126	8558	8863	9273
	ASR_YLLs	895	873	819	848	887
	YLDs (Crude)	135	132	130	130	133
	ASR_YLDs	12.9	12.7	12.4	12.4	12.7

SNNPs	DALYs (Crude)	38,176	37,040	36,149	39,671	43,050
	ASR_DALYs	1542	1496	1460	1602	1739
	YLLs (Crude)	37,406	36,270	35,377	38,893	42,248
	ASR_YLLs	1511	1465	1429	1571	1707
	YLDs (Crude)	770	770	772	778	803
	ASR_YLDs	31.1	31.2	31.2	31.4	32.4

Tigray	DALYs (Crude)	14,046	13,937	13,438	13,790	14,435
	ASR_DALYs	1092	1084	1045	1072	1122
	YLLs (Crude)	13,805	13,700	13,202	13,554	14,191
	ASR_YLLs	1073	1065	1027	1054	1103
	YLDs (Crude)	241	237	235	236	243
	ASR_YLDs	18.7	18.4	18.3	18.4	18.9

## 4. Discussion

HCV infection remains an important public health concern in Ethiopia because of its contribution to chronic liver disease, cirrhosis, hepatocellular carcinoma, and liver‐related mortality [21]. Although HCV is incorporated into Ethiopia’s national hepatitis surveillance and reporting framework, differences in diagnostic capacity, testing access, healthcare utilization, and reporting completeness may influence the availability of empirical data across regions. These differences should be considered when interpreting GBD estimates, as regional variation may reflect both underlying epidemiological differences and variation in the evidence informing the models. Strengthening standardized surveillance, diagnosis, and reporting is therefore essential for improving HCV burden estimation and guiding elimination interventions [14].

In this study, HCV prevalence and incidence increased between 2019 and 2023, accompanied by increases in age‐standardized prevalence and incidence rates. These findings are consistent with Ethiopian studies reporting increasing HCV prevalence between 2021 and 2023 [22] and substantial prevalence in 2018 and 2020 [23]. A 6‐year retrospective study also reported a significant increase in HCV prevalence from 2008 to 2015 (P for trend = 0.001) [24]. Globally, an estimated 170 million people have HCV infection, with sub‐Saharan Africa among the regions with the highest prevalence [1]. The observed increase in Ethiopia may reflect ongoing transmission, improved detection, population growth and aging, improved survival, and differences in the availability of epidemiological data rather than transmission alone. Notably, the GBD estimates were substantially higher than the approximately 0.18% weighted anti‐HCV seroprevalence reported by a recent nationally representative EDHS‐based study and differed from 2016 EDHS estimates. These estimates are not directly interchangeable because EDHS measures population‐based anti‐HCV seroprevalence, whereas GBD produces modeled estimates using multiple data sources and assumptions. Differences in case definitions, diagnostic methods, population representation, and data availability may partly explain this discrepancy, underscoring the need to triangulate GBD estimates with nationally representative serological and virological surveillance data.

HCV‐related deaths increased during the study period, while the crude mortality rate declined slightly and the ASMR increased. The apparent difference reflects the influence of population age structure on crude rates; therefore, the decline in crude mortality should not be interpreted as evidence of reduced HCV‐related mortality. The difference arises because crude mortality rates are influenced by the underlying age structure of the population, whereas ASRs adjust for differences in the population age structure and facilitate comparison across years. Therefore, the decrease in the crude mortality rate should not be interpreted as evidence of a decline in HCV‐related mortality. The increasing age‐standardized mortality is consistent with the substantial global burden of HCV, including approximately 0.54 million deaths and 15.3 million DALYs in 2019 [25]. Global HCV‐related death rates also increased between 1990 and 2019 [26]. Mortality is largely attributable to HCV‐related cirrhosis and hepatocellular carcinoma, which remain major causes of liver‐related deaths [27]. In Ethiopia, hepatitis C infection continues to contribute substantially to chronic liver disease and liver‐related mortality [1].

The overall HCV burden, measured by DALYs, increased during 2019–2023, with YLLs accounting for most DALYs and YLDs contributing comparatively little. This pattern indicates that premature mortality was the predominant component of health loss during the study period. The increase in age‐standardized mortality and DALY rates may reflect the long‐term consequences of chronic HCV infection, including progression to cirrhosis and hepatocellular carcinoma, which can occur years or decades after infection. This finding is consistent with evidence of increasing HCV‐related liver cancer DALYs [28]. Although age‐standardized DALY rates have declined in some settings, population growth and aging have maintained a substantial absolute HCV burden, particularly in low‐ and middle‐income countries [29]. The burden of viral hepatitis in Africa may also be underestimated because of gaps in diagnosis, surveillance, mortality ascertainment, and epidemiological data. Comparisons of HCV DALYs with hepatitis B, HIV, and malaria should therefore be interpreted within the GBD framework and in light of differences in disease ascertainment and available evidence. Strengthening testing, surveillance, and cause‐of‐death ascertainment remains important for better characterizing the burden. Similarly, YLLs increased between 2019 and 2023, with the age‐standardized YLL rate rising from 418 to 450 per 100,000. In contrast, YLDs increased only slightly, while the age‐standardized YLD rate remained stable at 7.3 per 100,000. The predominance of YLLs indicates that premature mortality contributed more to the HCV burden than disability, consistent with the continuing contribution of hepatitis‐related cirrhosis and hepatocellular carcinoma to premature mortality worldwide [30]. Limited access to curative antiviral therapy may also contribute to persistent HCV‐related morbidity and mortality in Ethiopia and other sub‐Saharan African countries [31].

Females had higher HCV prevalence and incidence than males throughout the study period, although incidence rates declined slightly in both sexes. This finding is consistent with an Ethiopian study reporting higher odds of HCV infection among females [32]. Similar female predominance has been reported elsewhere [33], whereas other studies have found higher HCV burden among males [34, 35]. Such inconsistencies may reflect differences in healthcare utilization, screening practices, behavioral exposures, and regional epidemiology. Prevalence increased with age and was highest among older adults, consistent with the chronic and often asymptomatic nature of HCV infection and the cumulative effect of exposure over time [36–39]. Older individuals are also more likely to develop HCV‐related cirrhosis and hepatocellular carcinoma [39]. Although absolute incident cases were observed across younger age groups, age‐specific incidence rates were highest among middle‐aged adults and increased most markedly among working‐age groups. Previous studies have similarly reported elevated HCV incidence among younger and middle‐aged adults [40, 41]. The generally lower incidence at older ages is consistent with the epidemiology of HCV, in which opportunities for new infection may decline with age [42].

Substantial regional variation was observed, with Oromia, Amhara, and SNNPs contributing large absolute numbers of prevalent, incident, and mortality cases. These patterns are consistent with previous Ethiopian studies reporting high HCV prevalence in Oromia and substantial numbers of cases in Amhara, southern Ethiopia, and central Ethiopia [2, 43]. The higher burden in these regions may partly reflect their large populations, differences in healthcare access and diagnostic capacity, historical exposure to healthcare‐related and community transmission pathways, and variation in epidemiological data available to the GBD modeling process. However, these factors were not directly measured in the present study and should therefore be considered plausible explanations rather than established causes. Absolute counts should also be interpreted alongside ASRs because population size strongly influences the number of events. The persistence of regional differences in standardized estimates suggests that population size alone does not explain the observed geographic heterogeneity. Previous GBD analyses have similarly documented substantial subnational differences in mortality, life expectancy, socioeconomic development, and disease burden across Ethiopia [44, 45].

The relatively small contribution of YLDs compared with YLLs further indicates that premature mortality was the major component of HCV‐related health loss. DALYs combine YLLs and YLDs to capture both premature death and nonfatal health loss [46]. The relatively stable YLD estimates do not imply an absence of morbidity but indicate that modeled mortality‐related health loss substantially exceeded disability‐related loss during the study period. Similar approaches have been used in Ethiopian burden‐of‐disease analyses, which demonstrate the importance of mortality‐related health loss [47]. The higher estimated burden in Oromia, Amhara, and SNNPs may partly reflect their large population sizes, differences in healthcare access and diagnostic capacity, historical exposure to healthcare‐related and community transmission pathways, and variation in the availability of epidemiological data used by the GBD modeling framework. However, because the present study did not directly measure these factors, we have presented them as plausible explanations rather than established causes. Differences in age‐standardized YLD rates between regions further highlight the importance of considering both absolute and standardized measures when interpreting subnational burden [41, 45].

### 4.1. Progress Toward WHO HCV Infection Elimination Targets

The findings should be interpreted in relation to the WHO 2030 HCV elimination targets, including an 80% reduction in incidence and 65% reduction in mortality from the 2015 baseline. The observed trends indicate that further reductions in HCV transmission and mortality are required to achieve these targets. Strengthening HCV testing, linkage to care, and access to curative DAA therapy is therefore essential. Given the relatively low HCV seroprevalence reported by recent nationally representative Ethiopian data, testing strategies should be guided by local epidemiology and prioritize populations with increased exposure risk rather than assuming that universal screening is appropriate in all settings. Prevention should also address modifiable exposure‐related behaviors through health education, safe injection and healthcare practices, blood safety, appropriate harm‐reduction interventions, and targeted testing. Because these factors were not directly measured in this study, interventions should be tailored to local epidemiological and health‐system contexts. Continued standardized surveillance is essential for monitoring progress and identifying geographic and population groups that remain underserved.

### 4.2. Policy Implications

The findings highlight the need to accelerate Ethiopia’s progress toward the WHO 2030 hepatitis C elimination targets. Priorities should include strengthening HCV case detection, linkage to care, and access to DAA treatment, particularly in underserved and higher‐burden populations. Given the relatively low HCV seroprevalence reported by recent nationally representative Ethiopian data, risk‐informed testing supported by strengthened surveillance may be more appropriate than population‐wide screening. Integrating HCV testing and treatment into HIV, TB, maternal, and primary healthcare services could further improve case identification and treatment access. Monitoring HCV incidence and mortality against the 2015 baseline and WHO 2030 targets is important for evaluating progress and guiding elimination strategies.

Although individual risk behaviors were not assessed, prevention should address blood‐borne exposure through health education, safe injection and healthcare practices, blood safety, appropriate harm‐reduction services, targeted testing, and linkage to curative treatment. These interventions should be tailored to local epidemiological and health‐system contexts to address regional differences in HCV burden.

### 4.3. Limitations of the Study

Despite providing valuable insights into the prevalence, incidence, and mortality trends of HCV infection in Ethiopia from 2019 to 2023, this study has some limitations that should be considered when interpreting the findings. First, the study relied primarily on GBD‐modeled estimates, which synthesize data from multiple sources, including vital registration, surveillance, and literature. While these models adjust for underreporting and misclassification, they may still differ from routine national surveillance counts. Second, the study did not collect data on coinfections, lifestyle factors, or socioeconomic determinants, which can significantly influence HCV infection outcomes. Third, regional comparisons may be affected by differences in diagnostic capacity, reporting systems, and healthcare infrastructure, which could lead to apparent disparities that partly reflect variation in data completeness rather than true differences in disease burden.

## 5. Conclusion

This study demonstrates a persistent and increasing burden of HCV infection in Ethiopia between 2019 and 2023. The number of prevalent HCV cases, incident infections, deaths, and DALYs increased over the study period, accompanied by rising age‐standardized prevalence, incidence, mortality, and DALY rates. The burden was heterogeneous across sex, age, and geographic regions. Females consistently experienced higher prevalence and incidence than males, while the greatest prevalence and incidence rates were concentrated among middle‐aged and older adults, particularly those aged 35–54 years. Oromia, Amhara, and SNNPs accounted for a substantial proportion of the national burden. These patterns may reflect differences in transmission, detection, population structure, healthcare access, and the availability of epidemiological data. Given the limitations of modeled estimates, these factors should be interpreted cautiously. Strengthening HCV prevention, testing, surveillance, linkage to care, and access to curative treatment is essential to support hepatitis C elimination in Ethiopia. The findings highlight the need to strengthen HCV infection prevention and control.

## Author Contributions

The corresponding author (Alemayehu Abate) was involved in the conception, design, and drafted the present manuscript and data analysis. Both authors (Alemayehu Abate and Melkamnesh Azage) were involved in analysis, interpretation of data, and in critically revising the manuscript for important intellectual content.

## Funding

No funding was given to support this study.

## Disclosure

The authors have reviewed and approved the manuscript for submission and publication.

## Ethics Statement

The study was exempt from institutional review board approval since it used aggregated, anonymized data from databases and publicly accessible sources. Every original source of data used in the GBD study complies with regional ethical standards.

## Conflicts of Interest

The authors declare no conflicts of interest.

## Supporting Information

Additional supporting information can be found online in the Supporting Information section.

## Supporting information


**Supporting Information** Annex 1: Supporting Table S1: Hepatitis C burden and temporal trends in Ethiopia, 2019–2023. Annex 2: Supporting Table S2: Age‐ and sex‐specific incidence and prevalence of hepatitis C in Ethiopia, 2019–2023. Annex 3: Supporting Table S3: Regional prevalence, incidence, deaths, and mortality rate trends of hepatitis C in Ethiopia, 2019–2023. Annex 4: Supporting Table S4: Regional YLLs, YLDs, and DALYs of hepatitis C in Ethiopia, 2019–2023.

## Data Availability

The data used in this study were obtained from the Global Burden of Disease (GBD) study, which is publicly available at https://www.healthdata.org/sites/default/files/projects/GBD/GBDcause_list. The specific GBD release, cause, measures, age groups, sex, years, and estimation parameters used for data extraction are reported in the Methods section. The extracted dataset generated in this article are included in the manuscript. The extracted data can be obtained from the corresponding author upon request Alemayehu Abate alexu2love@gmail.com.
