# Supplementary material for: National and Regional Burden of Hepatitis C in Ethiopia: A Secondary Analysis of the Global Burden of Disease Study 2023
Source: Can J Gastroenterol Hepatol. 2026 Oct 21;2026:2028285. doi: 10.1155/cjgh/2028285 (PMC13686396; doi:10.1155/cjgh/2028285)
Supplement: Supplementary file 1 [file CJGH-2026-2028285-s001.docx]

Annex 1: Supplementary Table S1: Hepatitis C Burden and Temporal Trends in Ethiopia, 2019–2023.

| Measure | 2019 | 2020 | 2021 | 2022 | 2023 |
| --- | --- | --- | --- | --- | --- |
| Prevalence | 1,525,506 (UI: 1,233,554–1,882,997) | 1,553,900 (UI: 1,255,440–1,919,659) | 1,584,972 (UI: 1,282,790–1,947,926) | 1,621,349 (UI: 1,299,684–1,989,025) | 1,653,765 (UI: 1,332,884–2,039,588) |
| Prevalence-Age-Standardized Rate | 1647 (UI 1,482 – 1,812) | 1671 (UI 1,504 – 1,838) | 1697 (UI 1,527 – 1,867) | 1724 (UI 1,552 – 1,896) | 1750 (UI 1,575 – 1,925 |
| Incidence | 188,080 (UI: 148,692–240,059) | 190,612 (UI: 150,139–243,710) | 193,744 (UI: 152,807–247,243) | 197,423 (UI: 154,520–251,829) | 200,228 (UI: 157,812–258,693) |
| Incidence-Age-Standardized Rate | 250 (UI 225 – 275) | 253 (UI 228 – 278) | 257 (UI 231 – 283) | 261 (UI 235 – 287) | 264 (UI 238 – 290) |
| Total Deaths | 7,019 (UI: 4,614–10,075) | 6,842 (UI: 4,478–9,893) | 6,675 (UI: 4,348–9,702) | 7,168 (UI: 4,613–10,573) | 7,623 (UI: 4,879–11,225) |
| Mortality Rate | 7.26 (UI 6.39 – 8.13) | 6.93 (UI 6.10 – 7.76) | 6.58 (UI 5.79 – 7.37) | 6.97 (UI 6.13 – 7.81) | 7.13 (6.27 – 7.99 |
| Mortality Rate0Age-Standardized Rate | 447(UI 393 – 501) | 428 (UI 376 – 480) | 438 (UI 385 – 491) | 466 (UI 410 – 522) | 500 (UI 440 – 560) |
| DALYs | 259,112 (UI: 165,317–381,194) | 253,836 (UI: 161,685–375,524) | 247,590 (UI: 157,050–372,777) | 268,648 (UI: 168,596–410,207) | 287,140 (UI: 176,866–437,025) |
| DALYs-Age-Standardized Rate | 426 (UI 383 – 469) | 419 (UI 377 – 461) | 408 (UI 367 – 449) | 434 (UI 391 – 477) | 457 (UI 411 – 503) |
| YLLs | 254,596 (UI: 161,246–375,627) | 249,338 (UI: 157,494–370,747) | 243,081 (UI: 153,070–368,101) | 264,086 (UI: 164,394–405,396) | 282,415 (UI: 172,238–432,101) |
| YLLs-Age-Standardized Rate | 418 (UI 376 – 460) | 412 (UI 371 – 453) | 401 (UI 361 – 441) | 427 (UI 384 – 470) | 450 (UI 405 – 495) |
| YLDs | 4,516 (UI: 2,681–7,170) | 4,498 (UI: 2,593–7,095) | 4,508 (UI: 2,601–7,181) | 4,562 (UI: 2,652–7,285) | 4,725 (UI: 2,763–7,530) |
| YLDs-Age-Standardized Rate | 7.3 (UI 6.6 – 8.0) | 7.2 (UI 6.5 – 7.9) | 7.2 (UI 6.5 – 7.9) | 7.1 (UI 6.4 – 7.8) | 7.3 (UI 6.6 – 8.0) |

Annex 2: Supplementary Table S2: Age and Sex Specific Incidence and Prevalence of Hepatitis C in Ethiopia, 2019–2023.

| Measure | Category | 2019 | 2020 | 2021 | 2022 | 2023 |
| --- | --- | --- | --- | --- | --- | --- |
| Prevalence (cases) | Female | 779,145 (UI: 634,869–951,012) | 796,193 (UI: 648,317–971,673) | 817,311 (UI: 667,804–991,516) | 840,858 (UI: 681,507–1,017,021) | 857,216 (UI: 700,453–1,046,893) |
|  | Male | 746,361 (UI: 598,685–931,985) | 757,707 (UI: 607,123–947,986) | 767,661 (UI: 614,986–956,410) | 780,491 (UI: 618,177–972,004) | 796,549 (UI: 632,431–992,695) |
| Prevalence rate | Female | 1,438 (UI: 1,435–1,441) | 1,437 (UI: 1,434–1,440) | 1,441 (UI: 1,438–1,444) | 1,450 (UI: 1,447–1,453) | 1,446 (UI: 1,443–1,449) |
|  | Male | 1,387 (UI: 1,384–1,390) | 1,378 (UI: 1,375–1,381) | 1,366 (UI: 1,363–1,369) | 1,357 (UI: 1,354–1,360) | 1,355 (UI: 1,352–1,358) |
|  | Male-to-female ratio | 0.965 (UI: 0.963–0.967) | 0.959 (UI: 0.957–0.961) | 0.948 (UI: 0.946–0.950) | 0.936 (UI: 0.934–0.938) | 0.937 (UI: 0.935–0.939) |
| Incidence (cases) | Female | 95,145 (UI: 76,309–119,983) | 96,645 (UI: 77,230–122,100) | 98,656 (UI: 79,060–124,440) | 100,938 (UI: 80,553–127,327) | 102,489 (UI: 81,956–131,675) |
|  | Male | 92,935 (UI: 72,383–120,076) | 93,967 (UI: 72,909–121,610) | 95,089 (UI: 73,746–122,803) | 96,485 (UI: 73,967–124,502) | 97,739 (UI: 75,856–127,018) |
| Incidence rate | Female | 176 (UI: 175–177) | 174 (UI: 173–175) | 175 (UI: 173–175) | 176 (UI: 173–175) | 173 (UI: 172–174) |
|  | Male | 173 (UI: 172–174) | 171 (UI: 170–172) | 169 (UI: 168–170) | 168 (UI: 167–169) | 166 (UI: 165–167) |
|  | Male-to-female ratio | 0.984 (UI: 0.981–0.987) | 0.959 (UI: 0.956–0.962) | 0.972 (UI: 0.969–0.975) | 0.964 (UI: 0.961–0.967) | 0.962 (UI: 0.959–0.965) |
| Prevalence (cases) | <5 years | 16,125 (UI: 12,628–19,838) | 16,191 (UI: 12,600–20,011) | 16,279 (UI: 12,656–20,029) | 16,400 (UI: 12,731–20,318) | 16,493 (UI: 12,714–20,606) |
|  | 5–14 years | 127,034 (UI: 100,774–155,263) | 127,081 (UI: 100,848–156,242) | 127,382 (UI: 101,246–155,628) | 127,961 (UI: 100,105–154,798) | 128,272 (UI: 102,744–155,521) |
|  | 15–24 years | 226,373 (UI: 184,393–274,856) | 228,959 (UI: 186,990–277,989) | 231,757 (UI: 187,852–281,916) | 234,907 (UI: 189,735–285,417) | 237,632 (UI: 191,224–287,933) |
|  | 25–34 years | 263,994 (UI: 214,514–322,555) | 269,399 (UI: 218,764–330,478) | 275,699 (UI: 223,133–335,958) | 282,656 (UI: 226,864–345,555) | 288,171 (UI: 230,902–355,194) |
|  | 35–44 years | 268,545 (UI: 215,942–340,072) | 275,458 (UI: 220,380–348,381) | 283,175 (UI: 228,073–355,161) | 291,572 (UI: 232,184–363,660) | 299,162 (UI: 239,586–377,267) |
|  | 45–54 years | 235,923 (UI: 190,608–294,570) | 242,162 (UI: 194,705–302,171) | 249,632 (UI: 200,845–309,403) | 258,080 (UI: 205,966–318,650) | 265,386 (UI: 213,680–331,872) |
|  | 55+ years | 387,512 (UI: 314,694–475,845) | 394,650 (UI: 321,153–484,388) | 401,049 (UI: 328,986–489,831) | 409,774 (UI: 332,099–500,627) | 418,649 (UI: 342,034–511,194) |
| Prevalence rate | <5 years | 215.0 (UI: 211.7–218.3) | 215.9 (UI: 212.6–219.2) | 217.1 (UI: 213.7–220.4) | 218.7 (UI: 215.3–222.0) | 219.9 (UI: 216.6–223.3) |
|  | 5–14 years | 705.7 (UI: 701.7–709.8) | 706.0 (UI: 702.0–710.0) | 707.7 (UI: 703.7–711.7) | 710.9 (UI: 706.9–714.9) | 712.6 (UI: 708.6–716.6) |
|  | 15–24 years | 2,791 (UI: 2,780–2,802) | 2,822 (UI: 2,811–2,833) | 2,856 (UI: 2,845–2,867) | 2,895 (UI: 2,884–2,906) | 2,930 (UI: 2,919–2,941) |
|  | 25–34 years | 3,867 (UI: 3,853–3,881) | 3,945 (UI: 3,931–3,959) | 4,038 (UI: 4,024–4,052) | 4,140 (UI: 4,125–4,155) | 4,224 (UI: 4,209–4,239) |
|  | 35–44 years | 5,048 (UI: 5,031–5,065) | 5,178 (UI: 5,161–5,195) | 5,323 (UI: 5,305–5,341) | 5,481 (UI: 5,463–5,499) | 5,628 (UI: 5,609–5,647) |
|  | 45–54 years | 6,682 (UI: 6,655–6,709) | 6,859 (UI: 6,832–6,886) | 7,070 (UI: 7,042–7,098) | 7,309 (UI: 7,280–7,338) | 7,515 (UI: 7,486–7,544) |
|  | 55+ years | 4,079 (UI: 4,066–4,092) | 4,154 (UI: 4,141–4,167) | 4,221 (UI: 4,208–4,234) | 4,313 (UI: 4,299–4,327) | 4,407 (UI: 4,393–4,421) |
| Incidence (cases) | <5 years | 16,012 (UI: 13,049–19,179) | 16,063 (UI: 13,014–19,370) | 16,131 (UI: 13,098–19,289) | 16,272 (UI: 12,795–19,534) | 16,428 (UI: 13,205–19,555) |
|  | 5–14 years | 35,821 (UI: 30,131–42,551) | 35,832 (UI: 29,961–42,758) | 35,939 (UI: 30,011–42,718) | 36,138 (UI: 29,906–43,050) | 36,278 (UI: 30,285–43,643) |
|  | 15–24 years | 34,920 (UI: 28,669–43,431) | 35,269 (UI: 28,854–43,783) | 35,673 (UI: 29,287–44,316) | 36,114 (UI: 29,471–45,064) | 36,451 (UI: 29,810–46,086) |
|  | 25–34 years | 30,305 (UI: 23,399–40,304) | 30,835 (UI: 23,809–40,969) | 31,511 (UI: 24,381–42,123) | 32,263 (UI: 24,882–42,484) | 32,842 (UI: 25,350–44,068) |
|  | 35–44 years | 28,561 (UI: 20,999–40,007) | 29,165 (UI: 21,413–40,788) | 29,891 (UI: 21,945–41,550) | 30,695 (UI: 22,418–42,330) | 31,440 (UI: 23,223–44,619) |
|  | 45–54 years | 23,484 (UI: 17,515–31,288) | 24,078 (UI: 17,863–32,252) | 24,844 (UI: 18,495–33,140) | 25,699 (UI: 19,142–34,547) | 26,181 (UI: 19,716–35,457) |
|  | 55+ years | 18,977 (UI: 14,931–23,299) | 19,369 (UI: 15,225–23,791) | 19,755 (UI: 15,590–24,107) | 20,242 (UI: 15,906–24,821) | 20,608 (UI: 16,223–25,264) |
| Incidence rate | <5 years | 213 (UI: 210–216) | 214 (UI: 211–217) | 215 (UI: 212–218) | 217 (UI: 214–220) | 219 (UI: 216–222) |
|  | 5–14 years | 413 (UI: 409–417) | 413 (UI: 409–417) | 415 (UI: 411–419) | 418 (UI: 414–422) | 421 (UI: 417–425) |
|  | 15–24 years | 429 (UI: 425–433) | 433 (UI: 429–437) | 438 (UI: 434–442) | 443 (UI: 439–447) | 447 (UI: 443–451) |
|  | 25–34 years | 443 (UI: 439–447) | 451 (UI: 447–455) | 461 (UI: 457–465) | 472 (UI: 468–476) | 480 (UI: 476–484) |
|  | 35–44 years | 537 (UI: 532–542) | 548 (UI: 543–553) | 562 (UI: 557–567) | 577 (UI: 572–582) | 592 (UI: 587–597) |
|  | 45–54 years | 660 (UI: 654–666) | 676 (UI: 670–682) | 698 (UI: 692–704) | 722 (UI: 716–728) | 735 (UI: 729–741) |
|  | 55+ years | 200 (UI: 197–203) | 204 (UI: 201–207) | 208 (UI: 205–211) | 213 (UI: 210–216) | 217 (UI: 214–220) |

Annex 3: Supplementary Table S3: Regional Prevalence, Incidence, Deaths and Mortality rate Trends of Hepatitis C in Ethiopia, 2019–2023.

| Regions | Metrix | 2019 | 2020 | 2021 | 2022 | 2023 |
| --- | --- | --- | --- | --- | --- | --- |
| Addis Ababa | Prevalence | 60158 (UI: 47301 - 75346) | 60839 (UI: 47419 - 76835) | 61235 (UI: 48179 - 76843) | 61968 (UI: 48161 - 78449) | 62696 (UI: 49092 - 79782) |
|  | Incidence | 6655 (UI: 4934 - 8864) | 6683 (UI: 4916 - 8979) | 6689 (UI: 5019 - 8876) | 6714 (UI: 4934 - 9012) | 6719 (UI: 4937 - 9080) |
|  | Deaths (Total Number) | 393 (UI: 251 - 595) | 384 (UI: 223 - 614) | 387 (UI: 186 - 677) | 403 (UI: 239 - 649) | 425 (UI: 253 - 688) |
|  | Mortality rate | 306 (UI: 194 - 464) | 291 (UI: 167 - 469) | 292 (UI: 135 - 516) | 298 (UI: 175 - 481) | 306 (UI: 181 - 496) |
| Afar | Prevalence | 28236 (UI: 22532 - 34842) | 28451 (UI: 22574 - 35282) | 28492 (UI: 22836 - 35065) | 28683 (UI: 22648 - 35692) | 28979 (UI: 22830 - 36177) |
|  | Incidence | 3860 (UI: 2970 - 5050) | 3873 (UI: 2933 - 5100) | 3873 (UI: 2977 - 4993) | 3888 (UI: 2898 - 5113) | 3904 (UI: 2914 - 5159) |
|  | Deaths (Total Number) | 118 (UI: 66 - 195) | 118 (UI: 63 - 200) | 116 (UI: 54 - 208) | 118 (UI: 67 - 199) | 126 (UI: 70 - 215) |
|  | Mortality rate | 312 (UI: 177 - 512) | 303 (UI: 164 - 507) | 296 (UI: 135 - 528) | 300 (UI: 174 - 498) | 312 (UI: 175 - 525) |
| Amhara | Prevalence | 360060 (UI: 287012 - 447614) | 364698 (UI: 287277 - 455988) | 371012 (UI: 295790 - 457846) | 378512 (UI: 297382 - 470029) | 384282 (UI: 304106 - 480606) |
|  | Incidence | 41130 (UI: 31659 - 53346) | 41398 (UI: 31301 - 54421) | 41903 (UI: 32135 - 53801) | 42534 (UI: 31849 - 55760) | 42928 (UI: 32401 - 56532) |
|  | Deaths (Total Number) | 1518 (UI: 968 - 2252) | 1482 (UI: 901 - 2230) | 1443 (UI: 830 - 2293) | 1539 (UI: 942 - 2318) | 1631 (UI: 1003 - 2451) |
|  | Mortality rate | 209 (UI: 132 - 312) | 198 (UI: 120 - 300) | 187 (UI: 106 - 301) | 196 (UI: 119 - 298) | 202 (UI: 122 - 308) |
| Benishangul-Gumuz | Prevalence | 20841 (UI: 16194 - 26134) | 21477 (UI: 16583 - 27116) | 22228 (UI: 17541 - 27901) | 23060 (UI: 18005 - 29142) | 23838 (UI: 18621 - 30063) |
|  | Incidence | 2798 (UI: 2083 - 3708) | 2868 (UI: 2106 - 3829) | 2951 (UI: 2209 - 3885) | 3044 (UI: 2242 - 4073) | 3128 (UI: 2313 - 4197) |
|  | Deaths (Total Number) | 89 (UI: 52 - 139) | 88 (UI: 49 - 143) | 88 (UI: 43 - 153) | 90 (UI: 53 - 149) | 95 (UI: 55 - 160) |
|  | Mortality rate | 286 (UI: 173 - 441) | 269 (UI: 150 - 431) | 255 (UI: 122 - 439) | 249 (UI: 148 - 403) | 251 (UI: 147 - 413) |
| Dire Dawa | Prevalence | 7163 (UI: 5641 - 9026) | 7256 (UI: 5634 - 9194) | 7333 (UI: 5758 - 9212) | 7442 (UI: 5739 - 9419) | 7532 (UI: 5900 - 9523) |
|  | Incidence | 844 (UI: 637 - 1117) | 849 (UI: 632 - 1134) | 854 (UI: 648 - 1127) | 862 (UI: 639 - 1159) | 866 (UI: 648 - 1152) |
|  | Deaths (Total Number) | 35 (UI: 21 - 55) | 35 (UI: 19 - 56) | 34 (UI: 17 - 57) | 35 (UI: 20 - 55) | 36 (UI: 21 - 59) |
|  | Mortality rate | 238 (UI: 144 - 372) | 227 (UI: 125 - 365) | 215 (UI: 104 - 367) | 216 (UI: 127 - 346) | 219 (UI: 125 - 356) |
| Gambella | Prevalence | 7010 (UI: 5506 - 8879) | 7162 (UI: 5578 - 9154) | 7281 (UI: 5732 - 9194) | 7441 (UI: 5745 - 9463) | 7600 (UI: 5866 - 9675) |
|  | Incidence | 895 (UI: 679 - 1189) | 907 (UI: 678 - 1221) | 918 (UI: 696 - 1221) | 933 (UI: 691 - 1257) | 942 (UI: 699 - 1266) |
|  | Deaths (Total Number) | 30 (UI: 17 - 48) | 30 (UI: 16 - 50) | 30 (UI: 13 - 53) | 31 (UI: 18 - 52) | 34 (UI: 19 - 59) |
|  | Mortality rate | 250 (UI: 148 - 397) | 242 (UI: 125 - 398) | 234 (UI: 98 - 418) | 238 (UI: 137 - 388) | 251 (UI: 141 - 423) |
| Harari | Prevalence | 5014 (UI: 3938 - 6253) | 5088 (UI: 3966 - 6393) | 5091 (UI: 4026 - 6354) | 5123 (UI: 4002 - 6455) | 5199 (UI: 4070 - 6566) |
|  | Incidence | 625 (UI: 459 - 841) | 631 (UI: 457 - 856) | 632 (UI: 468 - 845) | 634 (UI: 464 - 857) | 637 (UI: 461 - 865) |
|  | Deaths (Total Number) | 19 (UI: 11 - 28) | 18 (UI: 10 - 29) | 18 (UI: 8 - 31) | 19 (UI: 11 - 30) | 20 (UI: 12 - 32) |
|  | Mortality rate | 247 (UI: 150 - 371) | 229 (UI: 125 - 367) | 224 (UI: 99 - 400) | 235 (UI: 138 - 375) | 240 (UI: 138 - 381) |
| Oromia | Prevalence | 499373 (UI: 387545 - 630079) | 510220 (UI: 391678 - 647514) | 522221 (UI: 406990 - 657180) | 536293 (UI: 412435 - 683074) | 551992 (UI: 424697 - 706695) |
|  | Incidence | 65410 (UI: 48855 - 86341) | 66566 (UI: 49279 - 88670) | 67998 (UI: 51167 - 89308) | 69661 (UI: 51196 - 92952) | 71266 (UI: 53048 - 96177) |
|  | Deaths (Total Number) | 2444 (UI: 1494 - 3664) | 2378 (UI: 1422 - 3647) | 2316 (UI: 1276 - 3745) | 2550 (UI: 1529 - 3958) | 2716 (UI: 1605 - 4302) |
|  | Mortality rate | 265 (UI: 162 - 396) | 249 (UI: 149 - 381) | 236 (UI: 129 - 382) | 255 (UI: 153 - 393) | 262 (UI: 155 - 411) |
| Sidama | Prevalence | 66650 (UI: 53919 - 81947) | 68047 (UI: 54686 - 83921) | 69575 (UI: 56695 - 84967) | 71230 (UI: 57230 - 87662) | 72126 (UI: 57017 - 89523) |
|  | Incidence | 8551 (UI: 6738 - 10937) | 8679 (UI: 6753 - 11184) | 8831 (UI: 6977 - 11217) | 8997 (UI: 6928 - 11570) | 9063 (UI: 6947 - 11733) |
|  | Deaths (Total Number) | 323 (UI: 187 - 506) | 311 (UI: 173 - 497) | 297 (UI: 152 - 509) | 320 (UI: 181 - 522) | 338 (UI: 185 - 556) |
|  | Mortality rate | 326 (UI: 191 - 509) | 302 (UI: 168 - 484) | 280 (UI: 139 - 482) | 295 (UI: 166 - 479) | 300 (UI: 165 - 492) |
| Somali | Prevalence | 96221 (UI: 77110 - 118238) | 97855 (UI: 77718 - 120954) | 99188 (UI: 79410 - 121911) | 101059 (UI: 79487 - 125387) | 103545 (UI: 81890 - 128715) |
|  | Incidence | 12587 (UI: 9844 - 16032) | 12747 (UI: 9850 - 16453) | 12901 (UI: 10097 - 16402) | 13106 (UI: 9908 - 17080) | 13344 (UI: 10174 - 17377) |
|  | Deaths (Total Number) | 414 (UI: 244 - 648) | 409 (UI: 230 - 654) | 403 (UI: 195 - 679) | 419 (UI: 242 - 665) | 446 (UI: 252 - 732) |
|  | Mortality rate | 265 (UI: 158 - 412) | 252 (UI: 143 - 399) | 240 (UI: 116 - 404) | 247 (UI: 144 - 389) | 253 (UI: 144 - 412) |
| South West | Prevalence | 39795 (UI: 31986 - 48667) | 40133 (UI: 32002 - 49401) | 40414 (UI: 32650 - 49452) | 40828 (UI: 32496 - 50617) | 41344 (UI: 32598 - 50973) |
|  | Incidence | 5128 (UI: 4015 - 6494) | 5143 (UI: 3982 - 6555) | 5163 (UI: 4089 - 6520) | 5196 (UI: 4006 - 6726) | 5217 (UI: 3964 - 6760) |
|  | Deaths (Total Number) | 235 (UI: 136 - 363) | 227 (UI: 127 - 365) | 214 (UI: 108 - 362) | 220 (UI: 125 - 365) | 230 (UI: 123 - 384) |
|  | Mortality rate | 385 (UI: 225 - 589) | 363 (UI: 202 - 576) | 333 (UI: 164 - 560) | 339 (UI: 193 - 555) | 344 (UI: 187 - 567) |
| SNNPs | Prevalence | 242477 (UI: 197577 - 297173) | 248944 (UI: 200969 - 305648) | 255902 (UI: 208874 - 311168) | 263225 (UI: 211684 - 323784) | 267385 (UI: 215016 - 328882) |
|  | Incidence | 28924 (UI: 22706 - 37035) | 29503 (UI: 22957 - 38076) | 30161 (UI: 23700 - 38564) | 30856 (UI: 23744 - 40025) | 31168 (UI: 23979 - 40321) |
|  | Deaths (Total Number) | 994 (UI: 623 - 1479) | 962 (UI: 570 - 1474) | 942 (UI: 493 - 1556) | 1028 (UI: 625 - 1621) | 1111 (UI: 662 - 1746) |
|  | Mortality rate | 247 (UI: 155 - 366) | 229 (UI: 135 - 352) | 216 (UI: 110 - 362) | 229 (UI: 139 - 361) | 238 (UI: 142 - 374) |
| Tigray | Prevalence | 92509 (UI: 72945 - 116780) | 93731 (UI: 73114 - 119250) | 95001 (UI: 75058 - 118646) | 96487 (UI: 75699 - 121581) | 97247 (UI: 75209 - 123393) |
|  | Incidence | 10670 (UI: 8041 - 14077) | 10764 (UI: 7988 - 14378) | 10869 (UI: 8277 - 14266) | 10997 (UI: 8233 - 14579) | 11047 (UI: 8136 - 14810) |
|  | Deaths (Total Number) | 407 (UI: 252 - 628) | 401 (UI: 225 - 631) | 388 (UI: 189 - 649) | 395 (UI: 234 - 636) | 413 (UI: 239 - 666) |
|  | Mortality rate | 209 (UI: 128 - 323) | 200 (UI: 111 - 317) | 188 (UI: 89 - 318) | 187 (UI: 110 - 304) | 189 (UI: 108 - 308) |
| ASR-Prevalence | | | | | | |
|  | Addis Ababa | 4,958.0 (UI: 4,544.1 – 5,396.7) | 4,986.3 (UI: 4,563.2 – 5,438.0) | 5,026.3 (UI: 4,602.1 – 5,478.9) | 5,080.2 (UI: 4,647.1 – 5,544.1) | 5,106.4 (UI: 4,669.9 – 5,574.2) |
|  | Afar | 2,471.3 (UI: 2,279.0 – 2,680.3) | 2,496.5 (UI: 2,300.0 – 2,712.5) | 2,510.4 (UI: 2,312.5 – 2,727.9) | 2,531.8 (UI: 2,330.3 – 2,752.8) | 2,549.1 (UI: 2,344.8 – 2,773.0) |
|  | Amhara | 28,523.1 (UI: 26,238.2 – 30,948.1) | 28,729.6 (UI: 26,396.7 – 31,224.1) | 29,032.5 (UI: 26,663.4 – 31,557.5) | 29,390.4 (UI: 26,975.2 – 31,978.4) | 29,668.7 (UI: 27,238.2 – 32,272.5) |
|  | Benishangul-Gumuz | 1,900.3 (UI: 1,741.6 – 2,071.5) | 1,946.5 (UI: 1,783.0 – 2,125.1) | 1,999.5 (UI: 1,830.3 – 2,183.4) | 2,055.0 (UI: 1,879.6 – 2,245.3) | 2,104.9 (UI: 1,924.5 – 2,300.7) |
|  | Dire Dawa | 523.6 (UI: 480.7 – 570.5) | 526.1 (UI: 482.4 – 574.1) | 528.9 (UI: 485.0 – 577.0) | 532.0 (UI: 487.7 – 580.8) | 531.2 (UI: 486.9 – 579.8) |
|  | Gambella | 641.9 (UI: 591.1 – 696.5) | 648.1 (UI: 596.4 – 703.9) | 653.9 (UI: 601.5 – 710.6) | 658.6 (UI: 605.5 – 716.2) | 662.1 (UI: 608.7 – 719.9) |
|  | Harari | 404.3 (UI: 373.2 – 437.9) | 407.5 (UI: 375.9 – 441.8) | 408.1 (UI: 376.5 – 442.3) | 409.6 (UI: 377.8 – 444.2) | 412.1 (UI: 380.1 – 446.6) |
|  | Oromia | 42,981.6 (UI: 39,441.9 – 46,769.5) | 43,812.2 (UI: 40,176.0 – 47,731.9) | 44,708.1 (UI: 40,990.4 – 48,743.4) | 45,685.5 (UI: 41,841.8 – 49,842.1) | 46,693.0 (UI: 42,730.2 – 50,975.2) |
|  | Sidama | 6,813.7 (UI: 6,291.8 – 7,362.1) | 6,902.9 (UI: 6,371.2 – 7,466.2) | 7,005.4 (UI: 6,464.4 – 7,579.2) | 7,113.0 (UI: 6,560.0 – 7,701.0) | 7,159.0 (UI: 6,601.7 – 7,754.1) |
|  | Somali | 8,942.4 (UI: 8,192.0 – 9,752.3) | 9,092.6 (UI: 8,319.1 – 9,934.8) | 9,228.5 (UI: 8,440.5 – 10,090.3) | 9,395.8 (UI: 8,586.4 – 10,276.1) | 9,587.1 (UI: 8,756.9 – 10,491.7) |
|  | South West | 3,872.2 (UI: 3,588.9 – 4,172.2) | 3,898.2 (UI: 3,611.4 – 4,203.8) | 3,919.7 (UI: 3,631.4 – 4,226.9) | 3,945.8 (UI: 3,653.8 – 4,257.1) | 3,972.1 (UI: 3,677.5 – 4,285.0) |
|  | SNNPs | 21,820.6 (UI: 20,134.6 – 23,630.9) | 22,238.6 (UI: 20,498.9 – 24,113.8) | 22,694.3 (UI: 20,916.7 – 24,611.4) | 23,159.5 (UI: 21,338.2 – 25,115.0) | 23,349.7 (UI: 21,507.8 – 25,329.2) |
|  | Tigray | 7,981.8 (UI: 7,360.6 – 8,649.4) | 8,047.8 (UI: 7,414.1 – 8,730.8) | 8,109.7 (UI: 7,470.5 – 8,800.5) | 8,187.4 (UI: 7,537.7 – 8,891.0) | 8,225.9 (UI: 7,569.8 – 8,935.8) |
| ASR-Incidence | | | | | | |
|  | Addis Ababa | 558.1 (UI: 510.2 – 611.4) | 557.6 (UI: 508.6 – 612.9) | 555.0 (UI: 507.3 – 608.4) | 554.6 (UI: 506.0 – 609.3) | 553.9 (UI: 505.3 – 608.7) |
|  | Afar | 378.6 (UI: 345.8 – 415.3) | 380.5 (UI: 346.7 – 418.6) | 381.3 (UI: 347.9 – 419.1) | 383.0 (UI: 349.1 – 421.5) | 384.4 (UI: 350.0 – 423.0) |
|  | Amhara | 3,904.6 (UI: 3,619.8 – 4,198.2) | 3,919.6 (UI: 3,627.3 – 4,224.6) | 3,948.9 (UI: 3,654.7 – 4,257.4) | 3,992.1 (UI: 3,691.0 – 4,309.5) | 4,015.8 (UI: 3,713.9 – 4,337.6) |
|  | Benishangul-Gumuz | 294.5 (UI: 268.4 – 322.9) | 299.7 (UI: 273.0 – 329.2) | 306.1 (UI: 278.4 – 337.0) | 312.8 (UI: 284.0 – 344.7) | 319.0 (UI: 289.4 – 351.8) |
|  | Dire Dawa | 82.5 (UI: 75.9 – 89.8) | 82.4 (UI: 75.6 – 89.9) | 82.4 (UI: 75.7 – 89.8) | 82.6 (UI: 75.8 – 90.1) | 82.5 (UI: 75.7 – 90.0) |
|  | Gambella | 99.1 (UI: 91.6 – 107.3) | 99.6 (UI: 92.1 – 107.9) | 100.2 (UI: 92.5 – 108.6) | 100.7 (UI: 92.9 – 109.2) | 100.8 (UI: 92.9 – 109.3) |
|  | Harari | 60.2 (UI: 55.6 – 65.3) | 60.6 (UI: 55.9 – 65.8) | 60.5 (UI: 55.8 – 65.7) | 60.6 (UI: 55.9 – 65.8) | 60.9 (UI: 56.1 – 66.1) |
|  | Oromia | 6,590.2 (UI: 6,115.7 – 7,098.6) | 6,674.7 (UI: 6,189.6 – 7,198.9) | 6,785.8 (UI: 6,292.1 – 7,324.3) | 6,917.4 (UI: 6,408.7 – 7,471.2) | 7,069.5 (UI: 6,544.3 – 7,640.7) |
|  | Sidama | 968.5 (UI: 902.3 – 1,039.0) | 979.4 (UI: 911.0 – 1,052.2) | 993.2 (UI: 924.2 – 1,067.4) | 1,008.2 (UI: 937.2 – 1,084.5) | 1,012.5 (UI: 941.1 – 1,089.3) |
|  | Somali | 1,411.0 (UI: 1,294.3 – 1,537.1) | 1,425.1 (UI: 1,305.0 – 1,555.8) | 1,440.8 (UI: 1,319.2 – 1,573.4) | 1,460.7 (UI: 1,336.0 – 1,597.0) | 1,482.5 (UI: 1,355.7 – 1,621.0) |
|  | South West | 517.1 (UI: 482.3 – 554.4) | 519.0 (UI: 483.7 – 557.1) | 521.3 (UI: 485.9 – 559.8) | 524.4 (UI: 488.6 – 563.6) | 526.1 (UI: 490.0 – 565.4) |
|  | SNNPs | 3,125.9 (UI: 2,901.5 – 3,363.7) | 3,181.1 (UI: 2,949.8 – 3,428.3) | 3,242.8 (UI: 3,006.4 – 3,496.0) | 3,304.6 (UI: 3,061.5 – 3,567.0) | 3,331.5 (UI: 3,086.2 – 3,597.5) |
|  | Tigray | 1,165.6 (UI: 1,083.4 – 1,253.7) | 1,171.0 (UI: 1,087.4 – 1,261.1) | 1,176.5 (UI: 1,093.0 – 1,267.0) | 1,185.8 (UI: 1,100.0 – 1,278.5) | 1,189.1 (UI: 1,102.4 – 1,282.6) |
| ASR-Mortality Rate | | | | | | |
|  | Addis Ababa | 11.52 (95% UI: 10.40–12.64) | 11.24 (95% UI: 10.14–12.34) | 11.34 (95% UI: 10.24–12.44) | 11.80 (95% UI: 10.65–12.95) | 12.45 (95% UI: 11.27–13.63) |
|  | Afar | 8.35 (95% UI: 7.54–9.16) | 8.35 (95% UI: 7.54–9.16) | 8.21 (95% UI: 7.42–9.00) | 8.35 (95% UI: 7.54–9.16) | 8.92 (95% UI: 8.06–9.78) |
|  | Amhara | 10.14 (95% UI: 9.16–11.12) | 9.90 (95% UI: 8.94–10.86) | 9.64 (95% UI: 8.70–10.58) | 10.27 (95% UI: 9.28–11.26) | 10.87 (95% UI: 9.82–11.92) |
|  | Benishangul-Gumuz | 10.53 (95% UI: 9.52–11.54) | 10.41 (95% UI: 9.42–11.40) | 10.41 (95% UI: 9.42–11.40) | 10.65 (95% UI: 9.63–11.67) | 11.23 (95% UI: 10.15–12.31) |
|  | Dire Dawa | 9.55 (95% UI: 8.63–10.47) | 9.55 (95% UI: 8.63–10.47) | 9.27 (95% UI: 8.37–10.17) | 9.55 (95% UI: 8.63–10.47) | 9.87 (95% UI: 8.92–10.82) |
|  | Gambella | 8.93 (95% UI: 8.07–9.79) | 8.93 (95% UI: 8.07–9.79) | 8.93 (95% UI: 8.07–9.79) | 9.23 (95% UI: 8.34–10.12) | 10.12 (95% UI: 9.15–11.09) |
|  | Harari | 10.02 (95% UI: 9.06–10.98) | 9.50 (95% UI: 8.58–10.42) | 9.50 (95% UI: 8.58–10.42) | 10.02 (95% UI: 9.06–10.98) | 10.56 (95% UI: 9.54–11.58) |
|  | Oromia | 8.97 (95% UI: 8.10–9.84) | 8.72 (95% UI: 7.88–9.56) | 8.49 (95% UI: 7.67–9.31) | 9.35 (95% UI: 8.45–10.25) | 9.94 (95% UI: 8.98–10.90) |
|  | Sidama | 9.12 (95% UI: 8.24–10.00) | 8.80 (95% UI: 7.95–9.65) | 8.40 (95% UI: 7.59–9.21) | 9.06 (95% UI: 8.19–9.93) | 9.56 |
|  | Somali | 8.17 (UI 7.38–8.96) | 8.06 (UI 7.29–8.83) | 7.96 (UI 7.19–8.73) | 8.26 (UI 7.47–9.05) | 8.78 (UI 7.94–9.62) |
|  | South West | 8.62 (UI 7.79–9.45) | 8.32 (UI 7.52–9.12) | 7.85 (UI 7.09–8.61) | 8.07 (UI 7.29–8.85) | 8.45 (UI 7.64–9.26) |
|  | SNNPs | 12.35 (UI 11.17–13.53) | 11.95 (UI 10.81–13.09) | 11.70 (UI 10.58–12.82) | 12.83 (UI 11.60–14.06) | 13.87 (UI 12.55–15.19) |
|  | Tigray | 10.59 (UI 9.58–11.60) | 10.44 (UI 9.44–11.44) | 10.10 (UI 9.13–11.07) | 10.27 (UI 9.29–11.25) | 11.45 (UI 10.36–12.54) |

Annex 4: Supplementary Table S4: Regional YLLs and YLDs and DALYs of Hepatitis C in Ethiopia, 2019–2023.

| Region | Measure | 2019 | 2020 | 2021 | 2022 | 2023 |
| --- | --- | --- | --- | --- | --- | --- |
| Addis Ababa | DALYs (Crude) | 15,845 (95% UI: 15,598–16,092) | 15,548 (95% UI: 15,304–15,792) | 15,679 (95% UI: 15,434–15,924) | 16,528 (95% UI: 16,276–16,780) | 17,388 (95% UI: 17,130–17,646) |
|  | ASR_DALYs | 1,248 (95% UI: 1,229–1,267) | 1,225 (95% UI: 1,206–1,244) | 1,235 (95% UI: 1,216–1,254) | 1,302 (95% UI: 1,282–1,322) | 1,370 (95% UI: 1,349–1,391) |
|  | YLLs (Crude) | 15,625 (95% UI: 15,380–15,870) | 15,331 (95% UI: 15,088–15,574) | 15,468 (95% UI: 15,224–15,712) | 16,324 (95% UI: 16,074–16,574) | 17,176 (95% UI: 16,919–17,433) |
|  | ASR_YLLs | 1,231 (95% UI: 1,212–1,250) | 1,208 (95% UI: 1,189–1,227) | 1,218 (95% UI: 1,199–1,237) | 1,286 (95% UI: 1,266–1,306) | 1,353 (95% UI: 1,332–1,374) |
|  | YLDs (Crude) | 221 (95% UI: 192–250) | 218 (95% UI: 189–247) | 211 (95% UI: 183–239) | 204 (95% UI: 176–232) | 212 (95% UI: 183–241) |
|  | ASR_YLDs | 17.4 (95% UI: 15.1–19.7) | 17.2 (95% UI: 14.9–19.5) | 16.6 (95% UI: 14.4–18.8) | 16.1 (95% UI: 13.9–18.3) | 16.7 (95% UI: 14.5–18.9) |
| Afar | DALYs (Crude) | 4,811 (95% UI: 4,675–4,947) | 4,808 (95% UI: 4,672–4,944) | 4,801 (95% UI: 4,665–4,937) | 4,932 (95% UI: 4,794–5,070) | 5,288 (95% UI: 5,146–5,430) |
|  | ASR_DALYs | 958 (95% UI: 931–985) | 958 (95% UI: 931–985) | 957 (95% UI: 930–984) | 983 (95% UI: 955–1,011) | 1,054 (95% UI: 1,025–1,083) |
|  | YLLs (Crude) | 4,721 (95% UI: 4,587–4,855) | 4,719 (95% UI: 4,585–4,853) | 4,712 (95% UI: 4,578–4,846) | 4,843 (95% UI: 4,707–4,979) | 5,196 (95% UI: 5,055–5,337) |
|  | ASR_YLLs | 941 (95% UI: 914–968) | 940 (95% UI: 913–967) | 939 (95% UI: 912–966) | 965 (95% UI: 938–992) | 1,035 (95% UI: 1,007–1,063) |
|  | YLDs (Crude) | 90 (95% UI: 72–108) | 89 (95% UI: 71–107) | 88 (95% UI: 70–106) | 89 (95% UI: 71–107) | 92 (95% UI: 73–111) |
|  | ASR_YLDs | 17.9 (95% UI: 14.3–21.5) | 17.8 (95% UI: 14.2–21.4) | 17.6 (95% UI: 14.0–21.2) | 17.7 (95% UI: 14.1–21.3) | 18.3 (95% UI: 14.6–22.0) |
| Amhara | DALYs (Crude) | 50,825 (95% UI: 50,383–51,267) | 49,748 (95% UI: 49,311–50,185) | 48,315 (95% UI: 47,885–48,745) | 52,056 (95% UI: 51,609–52,503) | 55,470 (95% UI: 55,009–55,931) |
|  | ASR_DALYs | 1,523 (95% UI: 1,510–1,536) | 1,490 (95% UI: 1,477–1,503) | 1,447 (95% UI: 1,434–1,460) | 1,559 (95% UI: 1,546–1,572) | 1,662 (95% UI: 1,649–1,675) |
|  | YLLs (Crude) | 49,886 (95% UI: 49,448–50,324) | 48,823 (95% UI: 48,390–49,256) | 47,376 (95% UI: 46,949–47,803) | 51,104 (95% UI: 50,661–51,547) | 54,495 (95% UI: 54,037–54,953) |
|  | ASR_YLLs | 1,495 (95% UI: 1,482–1,508) | 1,463 (95% UI: 1,450–1,476) | 1,419 (95% UI: 1,406–1,432) | 1,531 (95% UI: 1,518–1,544) | 1,633 (95% UI: 1,620–1,646) |
|  | YLDs (Crude) | 939 (95% UI: 879–999) | 925 (95% UI: 866–984) | 939 (95% UI: 879–999) | 952 (95% UI: 891–1,013) | 975 (95% UI: 914–1,036) |
|  | ASR_YLDs | 28.1 (95% UI: 26.3–29.9) | 27.7 (95% UI: 25.9–29.5) | 28.0 (95% UI: 26.2–29.8) | 28.4 (95% UI: 26.6–30.2) | 29.1 (95% UI: 27.3–30.9) |
| Benishangul-Gumuz | DALYs (Crude) | 3,660 (95% UI: 3,541–3,779) | 3,621 (95% UI: 3,503–3,739) | 3,581 (95% UI: 3,464–3,698) | 3,702 (95% UI: 3,583–3,821) | 3,928 (95% UI: 3,805–4,051) |
|  | ASR_DALYs | 1,314 (95% UI: 1,271–1,357) | 1,300 (95% UI: 1,258–1,342) | 1,285 (95% UI: 1,243–1,327) | 1,329 (95% UI: 1,286–1,372) | 1,410 (95% UI: 1,365–1,455) |
|  | YLLs (Crude) | 3,593 (95% UI: 3,476–3,710) | 3,553 (95% UI: 3,436–3,670) | 3,511 (95% UI: 3,395–3,627) | 3,632 (95% UI: 3,514–3,750) | 3,855 (95% UI: 3,733–3,977) |
|  | ASR_YLLs | 1,290 (95% UI: 1,248–1,332) | 1,276 (95% UI: 1,234–1,318) | 1,261 (95% UI: 1,220–1,302) | 1,304 (95% UI: 1,262–1,346) | 1,384 (95% UI: 1,339–1,429) |
|  | YLDs (Crude) | 68 (95% UI: 52–84) | 69 (95% UI: 53–85) | 69 (95% UI: 53–85) | 70 (95% UI: 54–86) | 74 (95% UI: 57–91) |
|  | ASR_YLDs | 24.4 (95% UI: 18.7–30.1) | 24.7 (95% UI: 18.9–30.5) | 24.7 (95% UI: 18.9–30.5) | 25.1 (95% UI: 19.2–31.0) | 26.3 (95% UI: 20.1–32.5) |
| Dire Dawa | DALYs (Crude) | 1,334 (95% UI: 1,262–1,406) | 1,326 (95% UI: 1,254–1,398) | 1,275 (95% UI: 1,205–1,345) | 1,324 (95% UI: 1,252–1,396) | 1,388 (95% UI: 1,315–1,461) |
|  | ASR_DALYs | 1,045 (95% UI: 989–1,101) | 1,039 (95% UI: 983–1,095) | 999 (95% UI: 944–1,054) | 1,037 (95% UI: 981–1,093) | 1,088 (95% UI: 1,030–1,146) |
|  | YLLs (Crude) | 1,313 (95% UI: 1,242–1,384) | 1,306 (95% UI: 1,235–1,377) | 1,255 (95% UI: 1,185–1,325) | 1,304 (95% UI: 1,233–1,375) | 1,368 (95% UI: 1,295–1,441) |
|  | ASR_YLLs | 1,029 (95% UI: 973–1,085) | 1,023 (95% UI: 967–1,079) | 983 (95% UI: 929–1,037) | 1,021 (95% UI: 965–1,077) | 1,072 (95% UI: 1,014–1,130) |
|  | YLDs (Crude) | 20 (95% UI: 11–29) | 20 (95% UI: 11–29) | 20 (95% UI: 11–29) | 20 (95% UI: 11–29) | 20 (95% UI: 11–29) |
|  | ASR_YLDs | 15.7 (95% UI: 8.6–22.8) | 15.8 (95% UI: 8.7–22.9) | 15.5 (95% UI: 8.5–22.5) | 15.8 (95% UI: 8.7–22.9) | 15.7 (95% UI: 8.6–22.8) |
| Gambella | DALYs (Crude) | 1,235 (95% UI: 1,166–1,304) | 1,263 (95% UI: 1,193–1,333) | 1,251 (95% UI: 1,182–1,320) | 1,310 (95% UI: 1,239–1,381) | 1,444 (95% UI: 1,370–1,518) |
|  | ASR_DALYs | 1,098 (95% UI: 1,037–1,159) | 1,123 (95% UI: 1,061–1,185) | 1,112 (95% UI: 1,050–1,174) | 1,164 (95% UI: 1,100–1,228) | 1,283 (95% UI: 1,217–1,349) |
|  | YLLs (Crude) | 1,214 (95% UI: 1,146–1,282) | 1,241 (95% UI: 1,172–1,310) | 1,230 (95% UI: 1,161–1,299) | 1,289 (95% UI: 1,219–1,359) | 1,422 (95% UI: 1,348–1,496) |
|  | ASR_YLLs | 1,079 (95% UI: 1,019–1,139) | 1,104 (95% UI: 1,043–1,165) | 1,094 (95% UI: 1,033–1,155) | 1,146 (95% UI: 1,083–1,209) | 1,264 (95% UI: 1,198–1,330) |
|  | YLDs (Crude) | 21 (95% UI: 12–30) | 21 (95% UI: 12–30) | 21 (95% UI: 12–30) | 21 (95% UI: 12–30) | 22 (95% UI: 13–31) |
|  | ASR_YLDs | 18.7 (95% UI: 10.7–26.7) | 19.0 (95% UI: 10.9–27.1) | 18.5 (95% UI: 10.6–26.4) | 18.6 (95% UI: 10.7–26.5) | 19.2 (95% UI: 11.0–27.4) |
| Harari | DALYs (Crude) | 728 (95% UI: 675–781) | 704 (95% UI: 652–756) | 704 (95% UI: 652–756) | 752 (95% UI: 698–806) | 792 (95% UI: 737–847) |
|  | ASR_DALYs | 1,182 (95% UI: 1,096–1,268) | 1,143 (95% UI: 1,059–1,227) | 1,143 (95% UI: 1,059–1,227) | 1,220 (95% UI: 1,133–1,307) | 1,286 (95% UI: 1,197–1,375) |
|  | YLLs (Crude) | 705 (95% UI: 653–757) | 681 (95% UI: 630–732) | 682 (95% UI: 631–733) | 730 (95% UI: 677–783) | 769 (95% UI: 715–823) |
|  | ASR_YLLs | 1,144 (95% UI: 1,060–1,228) | 1,105 (95% UI: 1,023–1,187) | 1,108 (95% UI: 1,026–1,190) | 1,185 (95% UI: 1,099–1,271) | 1,248 (95% UI: 1,160–1,336) |
|  | YLDs (Crude) | 23 (95% UI: 14–32) | 23 (95% UI: 14–32) | 22 (95% UI: 13–31) | 22 (95% UI: 13–31) | 23 (95% UI: 14–32) |
|  | ASR_YLDs | 37.4 (95% UI: 22.8–52.0) | 37.7 (95% UI: 23.0–52.4) | 36.1 (95% UI: 22.0–50.2) | 36.2 (95% UI: 22.1–50.3) | 37.6 (95% UI: 22.9–52.3) |
| Oromia | DALYs (Crude) | 89,919 (95% UI: 89,331–90,507) | 88,075 (95% UI: 87,493–88,657) | 86,032 (95% UI: 85,457–86,607) | 96,002 (95% UI: 95,395–96,609) | 102,450 (95% UI: 101,823–103,077) |
|  | ASR_DALYs | 1,096 (95% UI: 1,089–1,103) | 1,073 (95% UI: 1,066–1,080) | 1,048 (95% UI: 1,041–1,055) | 1,170 (95% UI: 1,163–1,177) | 1,256 (95% UI: 1,249–1,263) |
|  | YLLs (Crude) | 88,471 (95% UI: 87,888–89,054) | 86,627 (95% UI: 86,050–87,204) | 84,571 (95% UI: 84,001–85,141) | 94,502 (95% UI: 93,900–95,104) | 100,876 (95% UI: 100,254–101,498) |
|  | ASR_YLLs | 1,079 (95% UI: 1,072–1,086) | 1,056 (95% UI: 1,049–1,063) | 1,031 (95% UI: 1,024–1,038) | 1,152 (95% UI: 1,145–1,159) | 1,237 (95% UI: 1,230–1,244) |
|  | YLDs (Crude) | 1,448 (95% UI: 1,373–1,523) | 1,449 (95% UI: 1,374–1,524) | 1,461 (95% UI: 1,386–1,536) | 1,500 (95% UI: 1,424–1,576) | 1,574 (95% UI: 1,496–1,652) |
|  | ASR_YLDs | 17.7 (95% UI: 16.8–18.6) | 17.7 (95% UI: 16.8–18.6) | 17.6 (95% UI: 16.7–18.5) | 17.8 (95% UI: 16.9–18.7) | 18.9 (95% UI: 18.0–19.8) |
| Sidama | DALYs (Crude) | 12,615 (95% UI: 12,395–12,835) | 12,237 (95% UI: 12,020–12,454) | 11,686 (95% UI: 11,474–11,898) | 12,718 (95% UI: 12,497–12,939) | 13,495 (95% UI: 13,267–13,723) |
|  | ASR_DALYs | 1,043 (95% UI: 1,025–1,061) | 1,012 (95% UI: 994–1,030) | 966 (95% UI: 949–983) | 1,052 (95% UI: 1,034–1,070) | 1,116 (95% UI: 1,097–1,135) |
|  | YLLs (Crude) | 12,350 (95% UI: 12,132–12,568) | 11,970 (95% UI: 11,755–12,185) | 11,421 (95% UI: 11,211–11,631) | 12,455 (95% UI: 12,236–12,674) | 13,231 (95% UI: 13,005–13,457) |
|  | ASR_YLLs | 1,021 (95% UI: 1,003–1,039) | 990 (95% UI: 972–1,008) | 944 (95% UI: 927–961) | 1,030 (95% UI: 1,012–1,048) | 1,094 (95% UI: 1,075–1,113) |
|  | YLDs (Crude) | 266 (95% UI: 234–298) | 267 (95% UI: 235–299) | 264 (95% UI: 232–296) | 262 (95% UI: 230–294) | 264 (95% UI: 232–296) |
|  | ASR_YLDs | 22.0 (95% UI: 19.3–24.7) | 22.2 (95% UI: 19.5–24.9) | 22.0 (95% UI: 19.3–24.7) | 21.8 (95% UI: 19.1–24.5) | 21.8 (95% UI: 19.1–24.5) |
| Somali | DALYs (Crude) | 16,433 (95% UI: 16,182–16,684) | 16,268 (95% UI: 16,019–16,517) | 15,991 (95% UI: 15,744–16,238) | 16,869 (95% UI: 16,614–17,124) | 17,980 (95% UI: 17,717–18,243) |
|  | ASR_DALYs | 1,162 (95% UI: 1,144–1,180) | 1,150 (95% UI: 1,132–1,168) | 1,131 (95% UI: 1,114–1,148) | 1,193 (95% UI: 1,175–1,211) | 1,272 (95% UI: 1,253–1,291) |
|  | YLLs (Crude) | 16,157 (95% UI: 15,908–16,406) | 15,992 (95% UI: 15,744–16,240) | 15,716 (95% UI: 15,470–15,962) | 16,592 (95% UI: 16,340–16,844) | 17,689 (95% UI: 17,428–17,950) |
|  | ASR_YLLs | 1,143 (95% UI: 1,125–1,161) | 1,131 (95% UI: 1,113–1,149) | 1,111 (95% UI: 1,094–1,128) | 1,173 (95% UI: 1,155–1,191) | 1,251 (95% UI: 1,232–1,270) |
|  | YLDs (Crude) | 275 (95% UI: 242–308) | 276 (95% UI: 243–309) | 275 (95% UI: 242–308) | 277 (95% UI: 244–310) | 290 (95% UI: 257–323) |
|  | ASR_YLDs | 19.5 (95% UI: 17.2–21.8) | 19.5 (95% UI: 17.2–21.8) | 19.7 (95% UI: 17.4–22.0) | 19.5 (95% UI: 17.2–21.8) | 20.5 (95% UI: 18.1–22.9) |
| South West | DALYs (Crude) | 9,486 (95% UI: 9,295–9,677) | 9,259 (95% UI: 9,070–9,448) | 8,688 (95% UI: 8,505–8,871) | 8,993 (95% UI: 8,807–9,179) | 9,405 (95% UI: 9,215–9,595) |
|  | ASR_DALYs | 908 (95% UI: 889–927) | 886 (95% UI: 868–904) | 832 (95% UI: 815–849) | 861 (95% UI: 843–879) | 900 (95% UI: 882–918) |
|  | YLLs (Crude) | 9,352 (95% UI: 9,162–9,542) | 9,126 (95% UI: 8,939–9,313) | 8,558 (95% UI: 8,376–8,740) | 8,863 (95% UI: 8,678–9,048) | 9,273 (95% UI: 9,084–9,462) |
|  | ASR_YLLs | 895 (95% UI: 877–913) | 873 (95% UI: 855–891) | 819 (95% UI: 802–836) | 848 (95% UI: 830–866) | 887 (95% UI: 869–905) |
|  | YLDs (Crude) | 135 (95% UI: 112–158) | 132 (95% UI: 109–155) | 130 (95% UI: 108–152) | 130 (95% UI: 108–152) | 133 (95% UI: 110–156) |
|  | ASR_YLDs | 12.9 (95% UI: 10.7–15.1) | 12.7 (95% UI: 10.5–14.9) | 12.4 (95% UI: 10.3–14.5) | 12.4 (95% UI: 10.3–14.5) | 12.7 (95% UI: 10.5–14.9) |
| SNNPs | DALYs (Crude) | 38,176 (95% UI: 37,793–38,559) | 37,040 (95% UI: 36,663–37,417) | 36,149 (95% UI: 35,776–36,522) | 39,671 (95% UI: 39,281–40,061) | 43,050 (95% UI: 42,644–43,456) |
|  | ASR_DALYs | 1,542 (95% UI: 1,527–1,557) | 1,496 (95% UI: 1,481–1,511) | 1,460 (95% UI: 1,445–1,475) | 1,602 (95% UI: 1,586–1,618) | 1,739 (95% UI: 1,722–1,756) |
|  | YLLs (Crude) | 37,406 (95% UI: 37,027–37,785) | 36,270 (95% UI: 35,896–36,644) | 35,377 (95% UI: 35,008–35,746) | 38,893 (95% UI: 38,506–39,280) | 42,248 (95% UI: 41,845–42,651) |
|  | ASR_YLLs | 1,511 (95% UI: 1,496–1,526) | 1,465 (95% UI: 1,450–1,480) | 1,429 (95% UI: 1,414–1,444) | 1,571 (95% UI: 1,555–1,587) | 1,707 (95% UI: 1,690–1,724) |
|  | YLDs (Crude) | 770 (95% UI: 716–824) | 770 (95% UI: 716–824) | 772 (95% UI: 718–826) | 778 (95% UI: 723–833) | 803 (95% UI: 747–859) |
|  | ASR_YLDs | 31.1 (95% UI: 28.9–33.3) | 31.2 (95% UI: 29.0–33.4) | 31.2 (95% UI: 29.0–33.4) | 31.4 (95% UI: 29.2–33.6) | 32.4 (95% UI: 30.2–34.6) |
| Tigray | DALYs (Crude) | 14,046 (95% UI: 13,814–14,278) | 13,937 (95% UI: 13,706–14,168) | 13,438 (95% UI: 13,211–13,665) | 13,790 (95% UI: 13,560–14,020) | 14,435 (95% UI: 14,199–14,671) |
|  | ASR_DALYs | 1,092 (95% UI: 1,074–1,110) | 1,084 (95% UI: 1,066–1,102) | 1,045 (95% UI: 1,027–1,063) | 1,072 (95% UI: 1,054–1,090) | 1,122 (95% UI: 1,104–1,140) |
|  | YLLs (Crude) | 13,805 (95% UI: 13,575–14,035) | 13,700 (95% UI: 13,471–13,929) | 13,202 (95% UI: 12,977–13,427) | 13,554 (95% UI: 13,326–13,782) | 14,191 (95% UI: 13,957–14,425) |
|  | ASR_YLLs | 1,073 (95% UI: 1,055–1,091) | 1,065 (95% UI: 1,047–1,083) | 1,027 (95% UI: 1,010–1,044) | 1,054 (95% UI: 1,036–1,072) | 1,103 (95% UI: 1,085–1,121) |
|  | YLDs (Crude) | 241 (95% UI: 211–271) | 237 (95% UI: 207–267) | 235 (95% UI: 205–265) | 236 (95% UI: 206–266) | 243 (95% UI: 212–274 |
|  | ASR_YLDs | 18.7 (95% UI:16.9-19.6) | 18.4 (95% UI:16.2-19.1) | 18.3 (95% UI:16.1-19.0) | 18.4 (95% UI:16.2-19.1) | 18.9 (95% UI:17.3-19.8) |

NB: SNNPs: Southern Nations, Nationalities, and Peoples, DALYs: Disability-Adjusted Life Years, YLLs: Years of Life Lost, YLDs: Years Lived with Disability.
